# Exploring imputation performance of phenotype-associated SNPs for forensic prediction models

**DOI:** 10.1038/s41598-026-63340-2

**Published:** 2026-07-22

**Authors:** Zehra Köksal, Andreas Tillmar

**Affiliations:** 1 Department of Biomedical and Clinical Sciences, Faculty of Health Sciences, Linköping University, 58183 Linköping, Sweden; 2 Department of Forensic Genetics and Forensic Toxicology, National Board of Forensic Medicine, 58758 Linköping, Sweden

**Keywords:** Forensic genetics, BEAGLE, Minor allele frequency, Facial prediction model, Linkage disequilibrium, Coding region imputation, Computational biology and bioinformatics, Diseases, Genetics

## Abstract

Imputing single nucleotide polymorphisms (SNPs) to expand genotyped SNP panels is commonly applied to study genotype-phenotype correlations in medical and population genetics but is largely unexplored in forensics. Forensic DNA phenotyping, i.e., the SNP-based phenotype prediction, can greatly benefit from imputing missing prediction model SNPs. Unfortunately, most imputation studies investigate the performance of random SNPs with limited focus on phenotype-associated SNPs. Here, we compare the imputation accuracy of SNPs leveraged in phenotype prediction models, phenotype-associated SNPs and all SNPs and explore the performance of phenotype prediction model, HIrisPlex-S, using imputed datasets. We confirm that the number and selection of SNPs in the genotype dataset and minor allele frequencies (MAFs) are major drivers of imputation call and error rates. Phenotypic SNPs show higher imputation error rates than randomly selected SNPs due to MAF differences. Further, we provide novel insights into accurate phenotype prediction for dense SNP panels of high linkage, frequent phenotypes in the reference panel, and lenient imputation thresholds due to the detrimental effect of missing genotypes on trait prediction compared to imputation errors. Overall, we show the importance of imputation performance studies for SNPs of interest and the high applicability of SNP imputations prior to phenotype prediction.

**Supplementary Information:**

The online version contains supplementary material available at 10.1038/s41598-026-63340-2.

## Introduction

A low-cost alternative to augment the size of an available single nucleotide polymorphism (SNP) panel is the imputation of SNPs. This in-silico method predicts alleles of loci that are absent in the dataset obtained from in-vitro experiments (i.e., in test samples), but that are present in a reference panel. Imputation tools utilize allele information on phased sequences (i.e., haplotypes) of the test sample to find the most probable shared segments between the test and reference panel samples. Within shared segments, ungenotyped alleles in the test sample can be inferred from the shared segment of the reference haplotypes. This is possible because SNPs on shared segments are non-randomly associated due to physical proximity or shared evolutionary history (linkage disequilibrium [LD]). Thus, the SNP allele information on such a haplotype implies the presence of additional SNP allele information on the same segment^1,2^. Consequently, genetic variants can be studied (with increased statistical power) that have not directly been genotyped or have only been genotyped in a small number of samples^3^. Additionally, genotype calls can be corrected and verified (genotype refinement)^4^.

In previous studies, several factors have been identified that impact the imputation performance: The size and composition of the reference panel have a great impact on the imputation accuracy. Larger panels tend to improve imputation accuracy due to increased probability to observe SNP patterns matching the test samples^1,2^. Most importantly, the imputation will benefit from a reference panel comprised of similar individuals as the test samples. This has recently prompted the implementation of customized population-specific or population-inclusive reference panels^5^. Secondly, increased number of SNPs in the test samples has shown to increase imputation accuracy, as more sites can match between test and reference panel samples^1,2^. Naturally, inaccuracies in the test and reference panel samples affect the imputation accuracy^2^. A study comparing the effect of drop-in and drop-out error types on the imputation accuracies, showed that both error types resulted in fewer imputed alleles and higher imputation error rates when compared to no introduced errors. Particularly, drop-in error types (turning homozygote genotypes to heterozygote genotypes) introduced the biggest imputation error rates^6^. Finally, rare alleles with low minor allele frequencies (MAFs) are observed less often in the reference panel, reducing the chance to find other SNPs linked with the rare allele, thus making them harder to impute^1,2^.

These imputation performance validations consider all SNPs that were genotyped^1,5,6^, which is reasonable for applications that make use of diverse SNPs, like in population genetics^7^. However, many studies are mainly interested in SNPs with functional consequences, like in medical genetics^8–10^. This includes the search for associations between causal SNPs and certain phenotypic traits, for example through genome wide association studies (GWAS)^11^. The identified trait-associated SNPs are essential to build accurate trait prediction models^12^, which also have a great potential in forensic genetics. In forensic genetics, forensic DNA phenotyping (FDP) describes the prediction of phenotypic traits based on genetic markers. FDP is helpful to provide investigative leads to narrow down the potential sample donors, when no suspect or reference sample is available^12^. Although genotype imputation would compensate for missing DNA markers caused by the crime scene samples’ low DNA quality and quantity or by targeted genotyping^12,13^, only few studies explore imputation in forensic genetics and none for FDP^6,13^. One reason for this may be the uncertainty about the imputation applicability and accuracy for FDP.

Indeed, we expect differences in the imputation of trait-associated SNPs compared to random SNPs, since trait-associated SNPs are mostly restricted to certain genomic regions. The genomic regions affect the size of LD blocks, which has a central role in the imputation performance^1,2,14,15^. Typically, LD blocks are more prominent in regions that escape recombination^14,16^, underwent natural selection^16–19^, translate to proteins or functional molecules^18^, and carry lower frequently-common SNPs^15,18^. Because of the prevalence of non-trait-associated SNPs compared to trait-associated SNPs^20^, the explicit analysis of the imputation performance of SNPs associated with traits is necessary to expand the current knowledge on SNP allele imputation and its applicability in medical and forensic genetics.

To address this, we utilize the concepts of SNP allele imputation to compare the genotype imputation performance for SNPs identified in GWAS (“PhenoSNPs“), SNPs selected for phenotype prediction models (“PredSNPs“) and all SNPs (“AllSNPs“). Here, we access data of 31 individuals from the 1000 Genomes Project of diverse biogeographic ancestries and diverse combinations of phenotypic traits according to our predictions. Finally, we assess the impact of imputation parameters on the accuracy of phenotype prediction models.

## Materials and methods

### Data collection and selection

Phased variant data of 2504 unrelated individuals from the 1000 Genomes Project phase 3 dataset^21^ were downloaded. Among these, 31 test samples with diverse phenotypic traits were selected based on (a) hair, eye and skin colour predictions using the HIrisPlex-S prediction model^22^ and (b) biogeographic ancestry (BGA) clusters estimated using the software STRUCTURE v2.3.4^23^.

For trait prediction (a), 39 out of the 41 SNPs used in the HIrisPlex-S prediction model (missing SNPs: rs312262906_A and rs201326893_A) were extracted and the phenotype probabilities were estimated using the HIrisPlex-S webservice (https://hirisplex.erasmusmc.nl/, accessed 05.03.25). The most probable traits per category were estimated using decision trees in accompanying papers^22,24^.

For prediction of BGA clusters (b), STRUCTURE^23^ was run for all 2504 samples using a burn-in of 10,000 and number of MCMC repetitions of 20,000 for 4 to 6 clusters (K) in 4 independent runs each. The most fitting number of clusters K was determined applying the software STRUCTURE harvester v0.6.93^25^ on all STRUCTURE output files. CLUMPP v1.1.2^26^ was applied using the largeKgreedy algorithm to combine the populations and individuals STRUCTURE output files for the most probable K.

For the following analyses, a diverse set of 31 individuals was selected covering diverse phenotypes of predicted traits from the HIrisPlex-S model and of diverse BGA clusters.

### Data cleaning

The 31 test samples were removed from the phased reference panel of 2504 samples, resulting in a reference panel of the remaining 2473 individuals.

The 1000 Genomes Project variant data of the 31 individuals were processed to remove sites with multiple entries of the same genomic locus with differing reference and alternative allele entries hinting towards ambiguous sites. Structural variants affecting more than 2 nucleotides (as reference or alternative allele) were removed, thus only retaining single nucleotide variants and short insertion and deletions. For conservative variant filtering, the program conform-gt (version from 24.05.2016, https://faculty.washington.edu/browning/conform-gt.html#introduction) was applied to the vcf files of the 31 individuals and the reference panel of 2,473 individuals to ensure that the vcf entries are consistent with the reference panel. Variants flagged as “ambiguous“ or “failing“ by conform-gt were removed from the vcf files, if the reference allele and/or any of the alternative alleles did not match 1 of the 4 possible nucleotides.

### SNP genotype imputation and performance statistics

For imputation performance tests, 7 subsets of the multisample vcf file of the 31 individuals were generated, representing the observed genotype data (from here on “preimputation datasets“). The preimputation datasets were filtered to contain either 70%, 30%, 10%, 5% or 1% randomly selected loci of the multisample vcf file for each chromosome 1 to 22 and chromosome X. Further, 2 preimputation datasets were generated to include only the 677,864 SNPs of the Infinium Global Screening Array-24 v3.0 BeadChip of Illumina Inc. (San Diego, CA, USA) (from here on “AncestryDNA panel“) and the 5446 SNPs of the FORCE panel^27^. These panels were selected due to their relevance and frequent application in genetic research^28^ and in forensic and investigative settings^29^.

These 7 preimputation datasets were unphased and used as input for imputation using BEAGLE v5.5^1^ with default parameters, except for burnin = 3, ap=true, gp=true, ne = 1,000,000, overlap = 4.0, and the HapMap GRCh37. The parameters were adapted to account for diverse and multi-ancestry populations, be applicable for sparse SNP panels and balance computational efficiency and imputation accuracy^1^. Genotypes were imputed if genotype or allele probability thresholds (0.99, 0.95, 0.9, 0.85, 0.5, 0.2, 0.1, and 0.01) were met and otherwise resulted in non-called genotypes.

To quantify the imputation performance, call rates describe the fraction of imputed and observed SNP genotypes divided by all SNP genotypes in the reference panel. The accuracy of imputed genotypes was presented as the squared correlation (allelic R^2^) between the allele dosage (minor allele) of the true genotype and the imputed genotype^30^. The imputation error rate was calculated as the fraction of incorrectly imputed SNP genotypes and all imputed SNP genotypes.

For all performance evaluations and throughout the article statistical significance was assessed at *p* < 0.05.

### SNP identification and characterisation

The imputation performance was tested for 1,164 SNPs utilized in 23 phenotypic trait prediction models^22,31–47^(“PredSNPs”; Supplementary Table 1). Additionally, 4663 SNPs were selected that were associated with phenotypic traits reported in single GWAS publications^31,48^ and in the GWAS catalogue (accessed 20.02.2025) by applying a list of 29 facial trait keywords that is comprehensive to capture all available SNPs associated with reported facial trait features (Supplementary Tables 2 and 3) (“PhenoSNPs“).

For comparison of SNP genotype imputation performance of coding versus noncoding region SNPs, the GENCODE database was accessed (version 37 for lift GRCh37).

### Predicting HIrisPlex-S phenotypes based on imputed datasets

The samples’ most probable eye, hair and skin colours were predicted using the HIrisPlex-S model for the above-described imputation datasets, where the expected traits were obtained by HIrisPlex-S predictions based on all 39 out of 41 available SNPs.

## Results

In the present study, we tested the imputation performance of SNPs connected with phenotypic traits. SNPs that are present in the 1000 Genomes Project dataset and leveraged in phenotype prediction models are referred to as “PredSNPs” and SNPs associated with phenotypic traits as “PhenoSNPs”. For comparison, all SNPs in the 1000 Genomes Project dataset are referred to as “AllSNPs”.

For these 3 SNP sets, we tested the impact of different imputation parameters and the number of SNPs in the preimputation dataset (i.e., observed genotype data) on the number of final SNPs and their imputation accuracy. The tested imputation parameters included conservative genotype and allele probability thresholds with cut-offs 0.95 and 0.99. The SNP density over all chromosomes in the preimputation dataset ranged from 30% missing data (21,424,640 SNPs) to 99% missing data (306,056 SNPs) including the 2 SNP panels AncestryDNA of ~ 98% missing data (618,383 SNPs) and the FORCE panel of ~ 99.9% missing data (4,612 SNPs). Finally, we assessed the impact of a range of strict to lenient genotype probability thresholds during imputation on the accuracy of the HIrisplex-S phenotype prediction model.

### Imputation call and accuracy rate depend on number and selection of SNPs in preimputation dataset

**Fig. 1:**
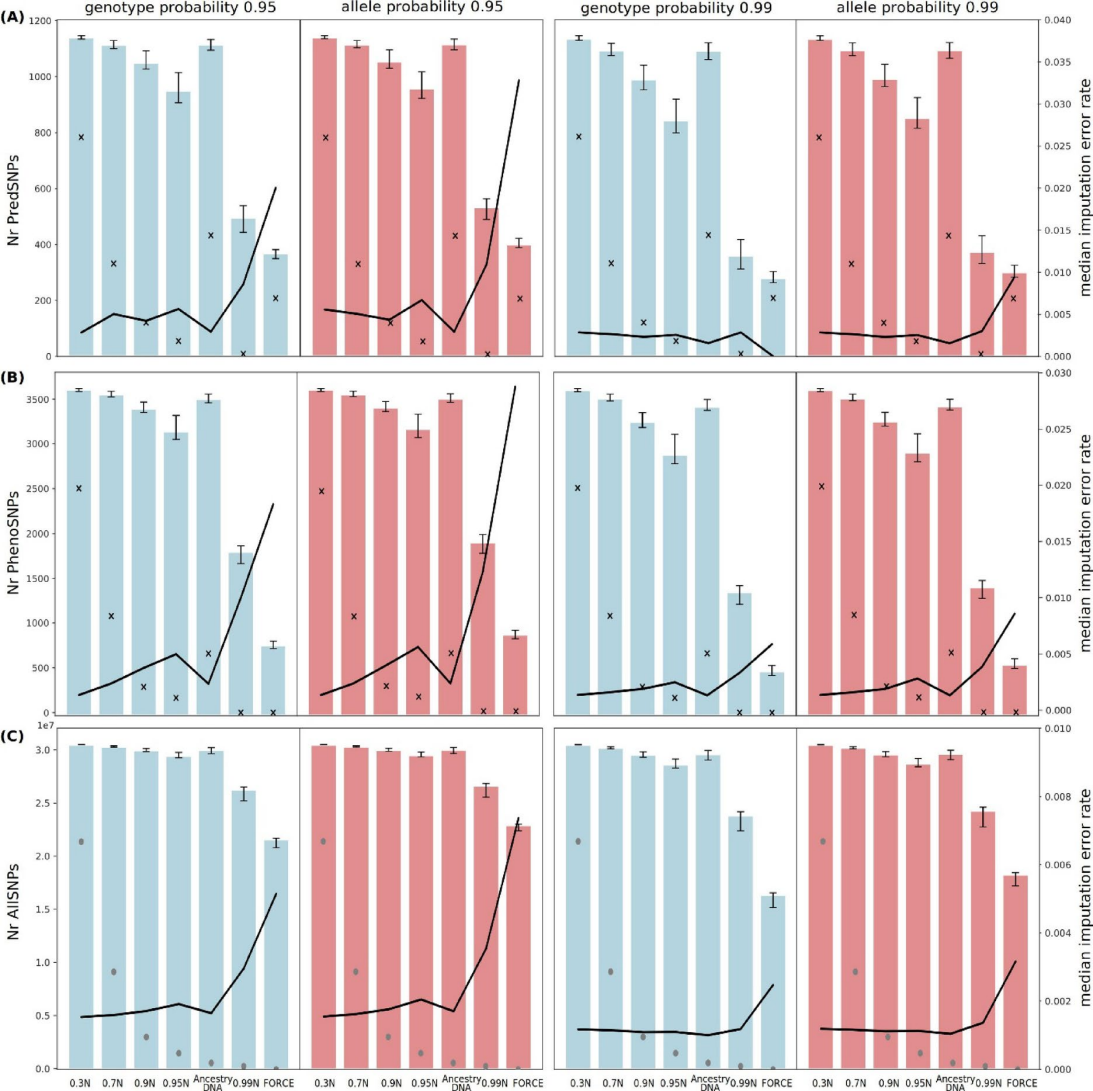
Imputation performance for (**A**) SNP genotypes from phenotype prediction models (PredSNPs; max SNPs = 1164), (**B**) SNP genotypes associated with phenotypic traits (PhenoSNPs; max SNPs = 4663), (**C**) and all imputed SNP genotypes (AllSNPs; max SNPs = 30,606,643). SNP genotypes were imputed in sets of the same 31 samples with increasing fractions of missing data from left to right on the x axes. The preimputation datasets correspond to missing data fractions of 30% (21,424,640 SNPs), 70% (9,181,980 SNPs), 90% (3,060,653 SNPs), 95% (1,530,320 SNPs), ~ 98% (AncestryDNA panel: 618,383 SNPs), 99% (306,056 SNPs) and ~ 99.9% (FORCE panel: 4612 SNPs). The number of SNPs in the preimputation datasets are marked as grey dots in subplot (**C**). Alleles were called using probability thresholds of 0.95 (left) or 0.99 (right) for genotypes (blue) or alleles (red). Bar heights correspond to median number of SNP genotypes after imputation and whiskers indicate minimum and maximum number of SNPs across all 31 samples per preimputation dataset. Line graphs corresponding to the secondary axes show median error rates of imputed SNP genotypes. Black crosses in subplots (**A**) and (**B**) indicate the number of PredSNPs and PhenoSNPs prior to imputation.

As shown in Fig. 1, the fewer SNPs in the preimputation dataset, the lower the imputation call rate and the higher the imputation error rate. The AncestryDNA panel presents an exception to this trend with a higher call and reduced error rate than expected given the number of SNPs in the preimputation dataset. Further, imputations using the FORCE panel and a genotype probability threshold of 0.99 for PredSNPs (Fig. 1A) resulted in an exceptionally low error rate (median: 0).

**Fig. 2:**
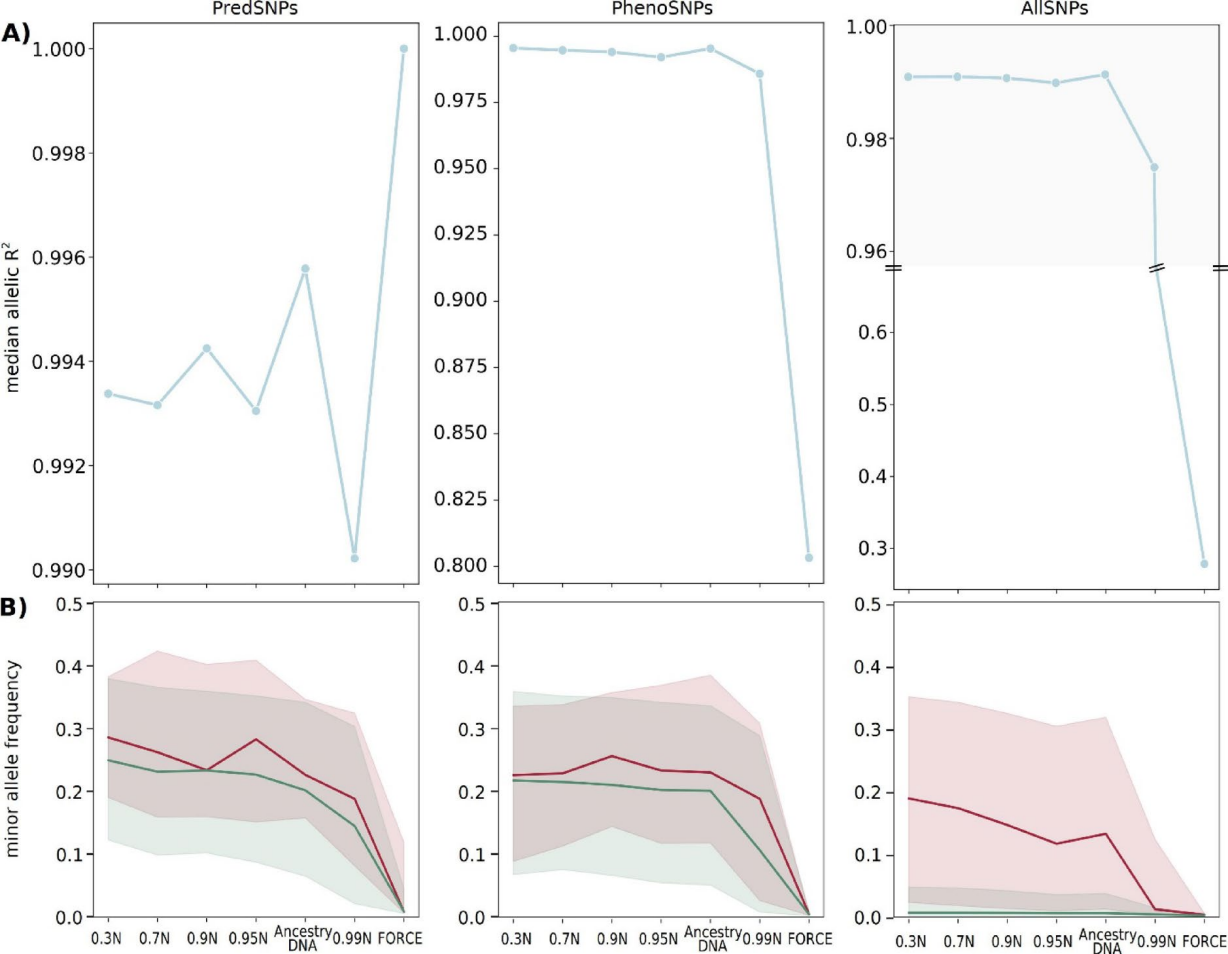
(**A**) Median allelic R^2^ across all 31 test samples for the 7 different preimputation datasets of decreasing panel density from left to right (x axes). (**B**) For correctly (in green) and incorrectly (in red) imputed SNP genotypes, the median (line) and 25th to 75th percentile (area) minor allele frequencies are shown for imputations of PredSNPs, PhenoSNPs and AllSNPs when using 7 different preimputation datasets of decreasing panel density from left to right (x axes) and a genotype threshold of 0.99 for imputation. In (**B**) the median minor allele frequencies of all imputed SNPs (correctly and incorrectly) align perfectly with the correctly imputed SNPs (green line).

Overall, similar imputation accuracy trends were observed when using the allelic R^2^ (Figs. 1 and 2A), which is the squared correlation of allele dosages of the minor allele between the true and imputed genotypes^30^. It ranges between 0 and 1, with values closer to 1 indicating higher imputation accuracies. For PredSNPs, the imputation error rate and allelic R^2^, have a very small variation across the 7 preimputation datasets. For PhenoSNPs and AllSNPs, both accuracy parameters decrease for smaller preimputation datasets (except for the AncestryDNA panel).

Figure 2B shows the median MAF of correctly (green) and incorrectly (red) imputed SNP genotypes that generally decreases with reducing preimputation dataset density. The MAF of correctly imputed SNP genotypes is generally lower than that of incorrectly imputed SNP genotypes. For AllSNPs this difference is most drastic.

Notably, the reported imputation inaccuracies also contain inaccuracies from the phasing of haplotypes that precedes the imputation. Such phasing inaccuracies are pronounced when using randomly selected sparse preimputation datasets and particularly affected PhenoSNPs (Supplementary Fig. 1).

### PredSNPs and PhenoSNPs tend to be more often incorrectly imputed compared to randomly selected SNPs

**Fig. 3:**
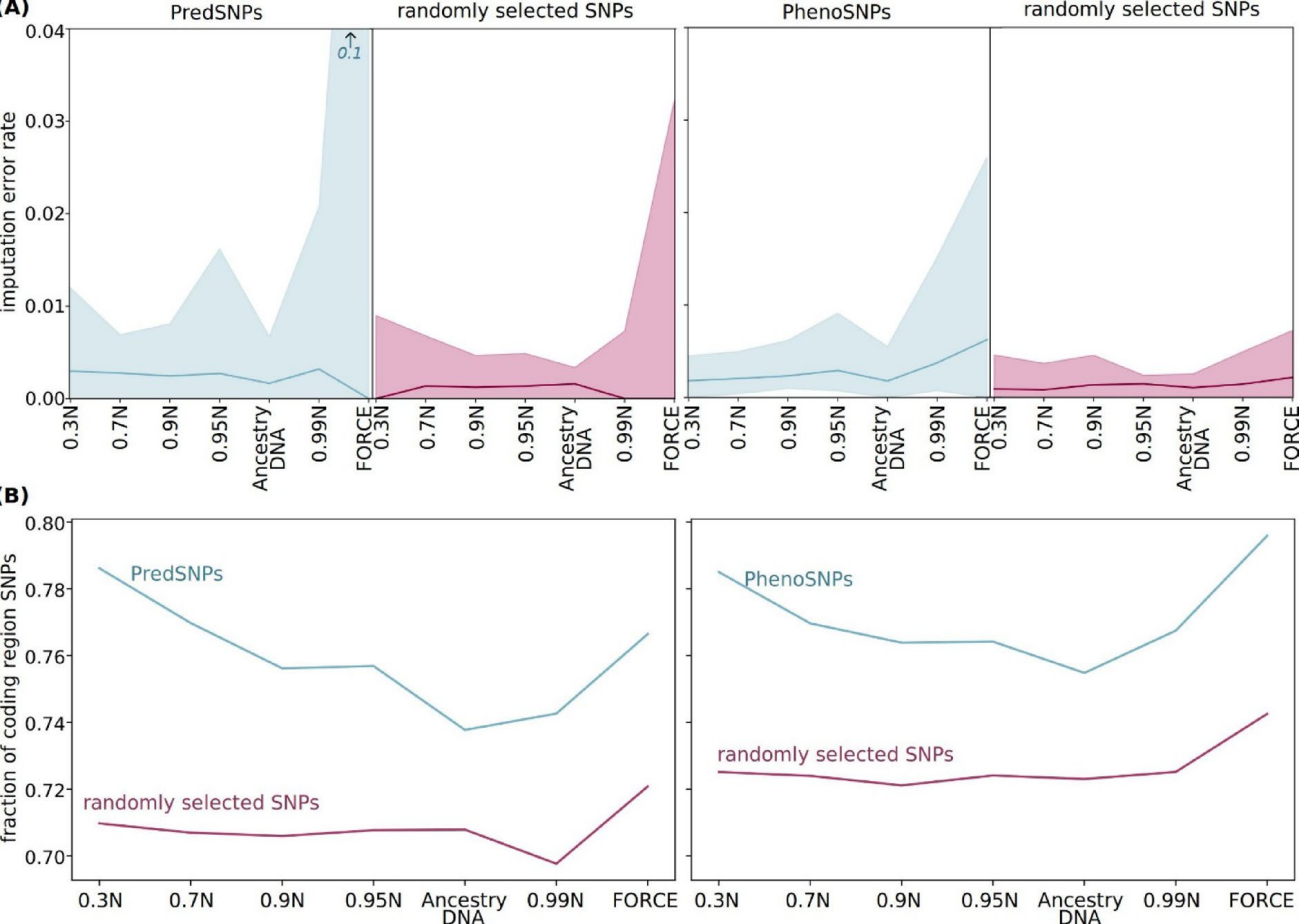
Comparison of imputation error rates of (**A**) PredSNPs and PhenoSNPs (blue) to an equal number of randomly selected SNPs (burgundy) for 7 preimputation datasets (x axes). Median imputation error rates are presented as darker blue or burgundy lines with the areas from the lowest to highest error rates represented in lighter shades. Imputation error rate for PredSNPs of FORCE panel have a maximum value of 0.1 (panel A, left). (**B**) Fraction of coding region SNPs across all imputed PredSNPs (blue) and randomly selected SNPs (burgundy) and PhenoSNPs (blue) and randomly selected SNPs (burgundy) for the 7 preimputation datasets.

Given the 10,000 to 26,000 times lower number of PredSNPs and PhenoSNPs compared to AllSNPs, we further explored imputation error rates after normalising the number of randomly selected SNPs in imputed datasets. We randomly selected SNPs in equal number to those exceeding the genotype probability cut-off of 0.99 for PredSNPs and PhenoSNPs, respectively. Figure 3A shows that the median imputation error rates of the same number of randomly selected SNPs are generally slightly lower than for the PredSNPs and PhenoSNPs. These differences are statistically significant as shown using a Wilcoxon signed-rank test to compare randomly selected SNPs with PredSNPs (Wilcoxon statistic = 2485.5, *p* = 2.0309e−10) and PhenoSNPs (Wilcoxon statistic = 2867, *p* = 2.3395e−17).

The fraction of coding region SNPs was consistently higher for the imputed PredSNPs and PhenoSNPs compared to the respective randomly selected SNPs (Fig. 3B). A Wilcoxon signed-rank test revealed a statistically significant difference between the two pairs of SNP sets (W = 0.0, *p* = 0.015625 for both).

### Size of preimputation dataset and abundance of phenotype in reference panel impact phenotype prediction accuracy

Finally, we aimed to analyse the effect of imputed datasets on the prediction accuracy of phenotypic traits and tested whether the prediction accuracy was correlated with the representation of the phenotype in the imputation reference panel. The HIrisPlex-S prediction model for predicting eye, skin and hair colour was used for this test. The phenotype predictions using the phased complete datasets of the 31 test and 2473 reference panel samples were considered as the “truth” (Supplementary Fig. 2).

**Fig. 4:**
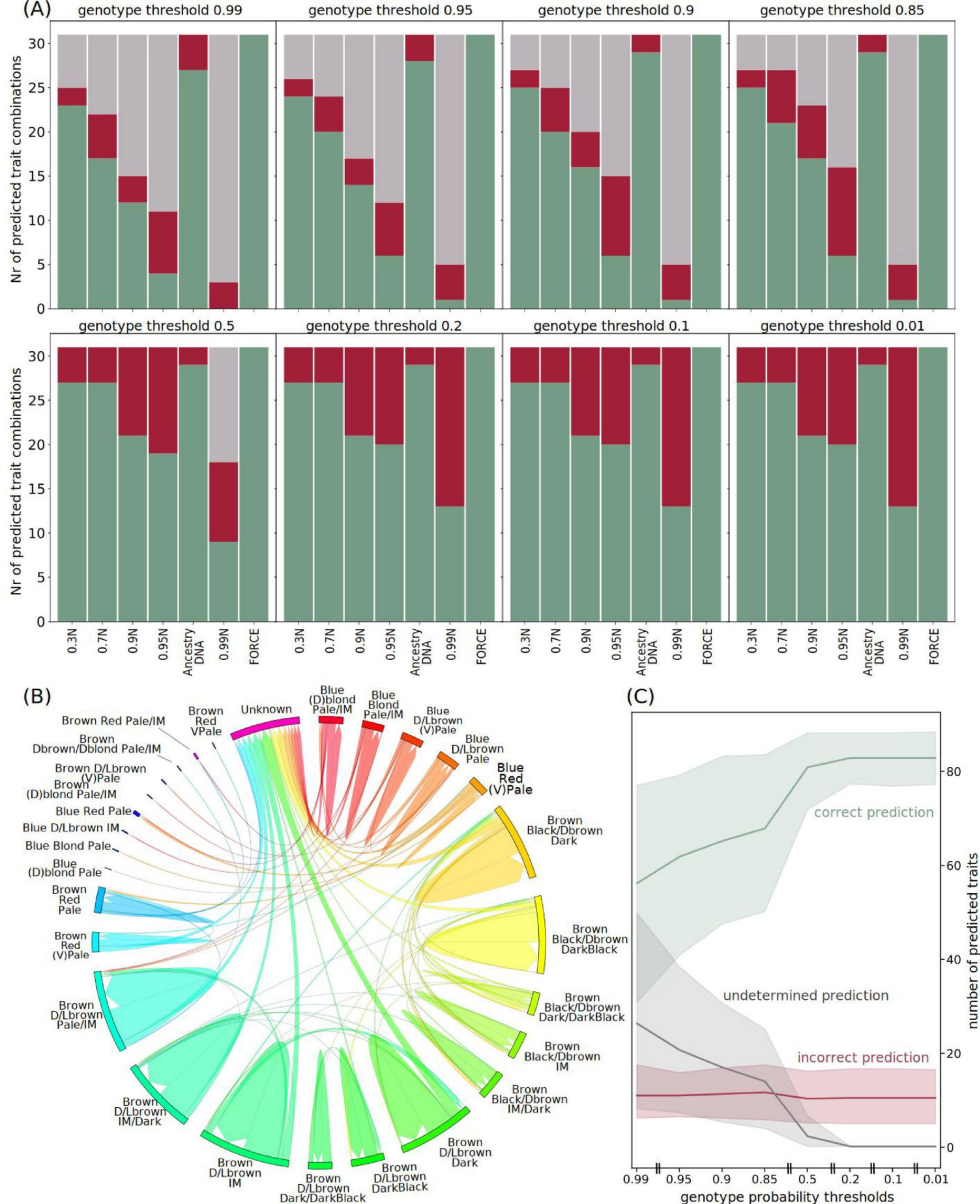
Performance of trait predictions. (**A**) Number of correctly (green) and incorrectly (red) predicted phenotypes (combination of eye, hair and skin colour) and inconclusive phenotypes (grey) of the 31 test samples in 7 preimputation datasets of decreasing number of SNPs prior to imputation. The subplots present prediction performances after imputation with decreasing genotype probability thresholds during imputation ranging from 0.99 (top left) to 0.01 (bottom right). The FORCE panel represents complete preimputation dataset without missing data. (**B**) Sankey plot with coloured sections presenting phenotypes with the true phenotypes represented as outgoing flows in the colour of the section and their predicted phenotypes as incoming flows. Predicted samples include 31 test samples and their 7 preimputation datasets and 8 imputation probability genotype thresholds. Phenotype labels include eye, hair and skin colour separated by spaces and are abbreviated with: D=Dark, L=Light, V=Very, IM=Intermediate. The plot was generated using pyCirclize v1.10. (**C**) Number of correctly (green), incorrectly (red) and undetermined (grey) trait predictions over all 31 samples. The lines represent the total number of traits of the 31 individuals averaged over the 6 different preimputation datasets (30% missing data to 99% missing data). The areas present 95% confidence intervals of the preimputation datasets. The trait prediction performance is presented for 8 different genotype probability thresholds with decreasing strictness from 0.99 to 0.01 along the x-axis. Note that the x-axis is not continuous.

After performing SNP genotype imputation in all 31 individuals using 7 unphased preimputation datasets, we re-predicted the HIrisPlex-S phenotypes. Generally, the number of correctly predicted phenotypes decreased with decreasing number of SNPs in the preimputation dataset (Fig. 4A). It is noteworthy that the prediction of phenotypes here refers to the aggregated phenotype of hair, eye and skin colour and does not distinguish between phenotypes with differing number and extent of mispredicted individual traits. This is particularly relevant given the pleiotropic nature of several loci of the HIrisPlex-S prediction model. The AncestryDNA and FORCE panels, which include 29 out of 39 and all 39 of the HIrisPlex-S SNPs, were exceptions to this observation.

Firstly, we investigated whether matching biogeographic ancestry (BGA) between test and reference panel samples during imputation impacted the HIrisPlex-S prediction accuracy. Due to the uneven number of test samples of the different BGAs (Supplementary Fig. 3), bootstrapping with replacement was applied as a resampling method to ensure even number of samples of the different BGAs. A negative correlation was found between (A) the number of reference panel samples with the same BGA as the test sample and (B) the frequency of the test sample’s inaccurate and inconclusive phenotype prediction across 7 preimputation datasets and 8 genotype probability thresholds (0.99 to 0.01). However, the correlation was statistically not significant (Pearson correlation = − 0.334, *p*-value = 5.827e−01).

Similarly, we evaluated the impact of the abundance of a test sample’s phenotype in the reference panel on the HIrisPlex-S prediction accuracy after bootstrapping. Considering all 7 preimputation datasets and 8 genotype probability thresholds, we observed a negative correlation between (A) the abundance of a phenotype in the reference panel and (B) the number of incorrect and inconclusive predictions of this phenotype (Supplementary Figs. 4–7). However, this correlation was statistically not significant (Pearson correlation = − 0.354, *p*-value = 1.493e−01).

Figure 4B presents the phenotype predictions contextualized by the samples’ true phenotypes across all sample datasets and imputation thresholds (see Supplementary Figs. 8 to 10 for specific imputation thresholds). The most frequently incorrectly or inconclusively predicted phenotype was the true phenotype blue eyes/red hair/very pale to pale skin, which was not correctly predicted in 160 bootstrapped predictions [BP] (Supplementary Table 4) and observed only once in the reference panel. The most frequent phenotype in the reference panel (brown eyes/dark brown to black hair/dark to black skin; *N* = 845) had the second fewest misidentifications (*N*=39BP). The similar phenotype brown eyes/dark to light brown hair/dark to black skin was most rarely misidentified (*N*=20BP) and observed 65 times in the reference panel (Supplementary Table 5). Phenotypes with many observations in the reference panel can also have substantial mispredictions (see Fig. 4B, Supplementary Table 5) but tend to be predicted more closely to the true phenotype (Supplementary Fig. 11).

### More lenient imputed SNP genotype calling thresholds give most accurate trait predictions

To further explore the impact of the SNP genotype imputation on the prediction of individual traits, we split the HIrisPlex-S SNPs into those that encode for the 3 different traits: 6 eye colour-defining SNPs, 22 hair colour-defining SNPs (2 of these are missing in the 1000 Genomes Project dataset), and 36 skin colour-defining SNPs. For the following analysis, the FORCE panel was removed since it contains all HIrisPlex-S SNPs. We applied a wide range of high-confidence to very permissive genotype probability thresholds (0.99, 0.95, 0.9, 0.85, 0.5, 0.2, 0.1 and 0.01) to accept the genotype with the highest probability above the selected threshold for the 6 different preimputation datasets with a focus on the higher-confidence thresholds that are commonly applied as cutoffs^6,49^. For the resulting imputed datasets, we then calculated the differences in allele dosage compared to the complete dataset, as presented in Supplementary Figs. 12–19.

As shown in Fig. 4C, decreasing genotype probability thresholds reduced undetermined trait predictions and increased the prediction call rate (Supplementary Fig. 20). Incorrect predictions rise slightly for moderately strict thresholds towards t = 0.85, then reduce with a minimum at t = 0.2 and beyond this, the performance remains stable (Supplementary Fig. 21). When considering only the predicted phenotypes, the average prediction accuracy increased for more lenient genotype thresholds, plateauing at t = 0.2 (Supplementary Fig. 22).

Multinomial logistic regressions were used to model the relationship of (A) missing genotypes and allele error rates with (B) the outcomes of correct, incorrect, and undetermined predictions. For stricter imputation call thresholds t > 0.5, missing SNP genotypes had a greater impact on the prediction outcomes than allele error rates. This impact of missing genotypes decreased for more lenient imputation call thresholds, ceasing for t ≤ 0.2 with no missing data.

## Discussion

In order to test the genotype imputation and the subsequent performance of the HIrisPlex-S prediction model, we used the 2504 unrelated Phase 3 samples of the 1000 Genomes Project^50^. These samples make up widely used imputation reference panels due to their wide geographic distribution from 26 worldwide human populations evenly distributed across the 5 major ancestry groups, Africans, Americans, East Asians, Europeans and South Asians^50^. To retain most of this diversity in the reference panel, while testing samples of diverse phenotypes and BGAs, we predicted the phenotypes of all 2504 samples using the HIrisPlex-S predictor. Afterwards, 31 samples of diverse phenotypes and BGAs were selected to represent frequent and rare phenotypes equally (Supplementary Figs. 2 and 3). The 31 samples were removed from the reference panel and subsampled to 7 preimputation datasets containing 30% to > 99% missing data, resulting in 217 datasets used for imputation.

A clear trend observed in this study is the reduced imputation call rate for smaller preimputation datasets, which aligns with earlier studies^6^. Since lower SNP densities correspond to fewer “anchoring” SNPs that can be identified in the reference panel, the number and size of determined linkage blocks with co-inherited SNPs is reduced as well. This further causes correctly imputed SNP genotypes to drop more than the total imputed SNP genotypes, which increases the imputation error rate. Accordingly, a negative correlation between SNP density in the preimputation dataset and imputation error rate was observed, as expected from the literature^6^(Fig. 1C). Similar accuracy patterns were observed when utilizing the allelic R^2^ that captures the correlation between true and imputed allele dosage and is therefore less impacted by allele frequencies than imputation error rates^30^. Consistent with the imputation error rate, the allelic R^2^ presents higher imputation accuracies for larger preimputation datasets that have fewer and smaller allele dosage differences between the true and imputed genotypes (Supplementary Figs. 23 and 24).

Imputations with the AncestryDNA panel lead to increased call and reduced error rate, because it includes a wide range of carefully selected human linkage SNPs with higher informative value per SNP than in the preimputation datasets of randomly selected SNPs. Using the FORCE panel and a genotype probability threshold of 0.99 for PredSNP led to exceptionally low imputation error rates (Fig. 1A), since the panel already includes 203 PredSNPs, leaving a median of only 77 SNPs to be imputed per sample. This immensely reduces the risk of observing wrongly imputed SNP genotypes.

Generally, imputation error rates and allelic R^2^ showed similar imputation accuracy trends. However, the imputation error rates showed higher imputation accuracies for FORCE panel-based imputations of AllSNPs compared to PhenoSNPs, while the allelic R^2^ showed the opposite trend (Fig. 1). This is because a smaller fraction of AllSNPs were wrongly imputed but had more often allele dosage differences of 2 between the true and imputed SNP genotype (Supplementary Fig. 23), when compared to PhenoSNPs that mainly had allele dosage differences of 1.

This contrast in allele dosage differences is likely due to AllSNPs’ much wider range of SNPs, which are mainly very rare (minor allele frequency [MAF] of 0.00699, see Supplementary Fig. 25)^51^, compared to PhenoSNPs. PhenoSNPs, like PredSNPs, have increased median MAFs of 0.215855 and 0.240815, respectively. Minor alleles of SNPs with low MAFs are more likely to be incorrectly imputed (Supplementary Fig. 26), because of limited allele observations in the reference panel^30,52^. In contrast, their major alleles are frequently observed in the reference panel and thus more likely correctly imputed, which increases imputation accuracy estimations due to the abundance of major alleles of SNPs with low MAF. Accordingly, the correct imputation of the major alleles of very rare SNPs, which are very abundant, are observed as correctly imputed SNPs having lower MAFs than incorrectly imputed SNPs (Fig. 2B).

The correct imputation of major alleles of rare SNPs also explains the significantly lower imputation error rates of two size-matched randomly selected SNP sets comprising of more rare SNPs (median MAFs 0.005791 [0.000200–0.5] and 0.005591 [0.000200–0.5], respectively) compared to PredSNPs and PhenoSNPs (median MAFs 0.219249 [0.000998–0.499401) and 0.191693 [0.000706–0.499800], respectively) (Fig. 3A, B). Alternatively, the imputation error rates could be compared between size- and MAF-matched imputed SNPs subsampled from AllSNPs, PredSNPs and PhenoSNPs.

Interestingly, compared to the randomly selected SNPs, PredSNPs and PhenoSNPs showed significantly higher fractions of coding region SNPs (Fig. 3), which are known to have increased linkage with each other compared to SNPs in non-coding regions^18^. This is because selection acts more restrictively on coding regions than non-coding regions, resulting in increased co-inheritance of coding region and nearby hitchhiking SNPs^18^. While LD blocks are larger in coding regions^18^, recombination occurs more frequently for regions outside of genes^53^. Thus, higher imputation accuracies would be expected for PredSNPs and PhenoSNPs compared to randomly selected SNPs. However, the differences in coding vs. non-coding region SNPs may have been too small to make up for the large MAF differences.

Notably, the presented imputation inaccuracies include the combined phasing and imputation inaccuracies. Reduced phasing errors were observed for SNP panels with increased linkage, i.e., the largest SNP panel and the two selected panels, AncestryDNA and FORCE panel, compared to panels with lower SNP density. For the highly linked panels, imputation inaccuracies are mainly attributed to errors during imputation instead of phasing. Among the panels with low SNP density, the phasing errors of PhenoSNPs propagated most to the imputation accuracy compared to PredSNPs and AllSNPs due to the high linkage of PhenoSNPs^14,16–19^. The impact of phasing errors on the imputation inaccuracies was lower for AllSNPs due to their lower linkage. Finally, phasing errors propagated least to the imputation of PredSNPs, as these SNPs were carefully selected to minimize linkage among themselves when establishing the prediction models.

Given the different weights of loci of the HIrisPlex-S prediction model on the trait prediction, and the pleiotropic effects of several loci, the phenotype prediction accuracy after imputation was explored. When testing how the performance of the prediction model, HIrisPlex-S, depends on the composition of the reference panel used for the imputation of its SNPs, we observed improved phenotype prediction performance for samples of BGAs more abundantly represented in the imputation reference panel. This was expected, since shared population history increases the chance of shared LD blocks and therefore improves the probability of choosing the correct haplotypes during imputation^16^. Accordingly, previous studies have shown that similar BGAs between test and reference panel samples improve imputation accuracy of general SNPs and have prompted the introduction of population-specific or ancestrally-matched reference panels for imputation^1,5,54^. Similarly, we observed that phenotypes represented more frequently in the imputation reference panel tend to be more often correctly predicted (Fig. 4B). This trend was not statistically significant, which was also indicated by variable prediction error rates among phenotypes with comparable frequency in the reference panel (Supplementary Table 4). The variable prediction error rates can be explained by the statistically significant positive correlation between the frequency of the phenotype in the reference panel and the similarity between the true and predicted phenotypes (Supplementary Fig. 11). This similarity was not represented in the prediction error rates. Taken together, this supports and extends previous findings that genetic similarity between test and reference panel samples is highly relevant for the accurate imputation of general and phenotype-associated SNPs. These findings however are limited by the modest sample size of 31 samples (and 217 preimputation datasets) resulting from our approach to represent rare and frequent phenotypes equally.

For the HIrisPlex-S prediction model, missing alleles resulting from strict to moderate genotype probability thresholds (0.99 ≥ t ≥ 0.5) have a greater impact on the phenotype prediction performance over incorrect alleles. Thus, the large fraction of missing SNPs when using strict genotype probability thresholds causes many unpredicted traits. Meanwhile, for moderately strict genotype probability thresholds, the number of correct phenotype predictions increases, as well as for incorrectly predicted phenotypes. This is most likely due to an unfavorable interaction of missing SNPs and incorrectly predicted SNPs. Lenient thresholds (t = 0.2) are most beneficial to improve the phenotype prediction performance using the HIrisPlex-S prediction model. This is likely because the information gained from available SNPs outweighs the negative implications from incorrectly imputed SNP genotypes (Fig. 4). Further, most incorrectly predicted phenotypes remain incorrectly predicted regardless of imputation thresholds. It is important to note that the added value of more lenient thresholds (t < 0.2) lies in accessing genotypes from low-confidence imputations for SNPs with more than two alleles (e.g., triallelic or tetraallelic SNPs). Multiallelic SNPs are less frequent and can result from technical artifacts, such as sequencing errors, and are therefore often excluded from investigations and prediction models. Selecting thresholds of t < 0.2 should therefore be considered with caution.

However, the optimal phenotype prediction performance using lenient genotype probability thresholds is specific to the HIrisPlex-S prediction model and might differ for other prediction tools. Alternative trait predictors may utilize structural variants, where failed allele calls may have a biological cause, such as deletions, that are frequently not in LD with their flanking SNPs. In these cases, the permutation of missing SNP calls can potentially obscure marker-trait associations^55^. This highlights the importance of testing the impact of SNP genotype imputation criteria on the prediction model performance to gain the most reliable results. In the future, a shift from predictors that use allele dosages to probabilistic classifiers that make use of genotype probabilities would offer a powerful and robust solution that effectively incorporates the genotype probability information.

The presented findings lay the groundwork for applying genotype imputation for forensic DNA phenotyping to provide investigative leads that can be combined with other scientific methods when repetitions of resource-intensive in-vitro experiments are not possible or successful.

## Supplementary Information

Below is the link to the electronic supplementary material.

Supplementary Material 1

Supplementary Material 2

## Data Availability

The variant data used in this study were obtained from the 1000 Genomes Project from the International Genome Sample Resource: https://www.internationalgenome.org/data.
