# Supplementary material for: Exploring imputation performance of phenotype-associated SNPs for forensic prediction models
Source: Sci Rep. 2026 Jul 22;16:22936. doi: 10.1038/s41598-026-63340-2 (PMC13392018; doi:10.1038/s41598-026-63340-2)
Supplement: Supplementary file 2 — Supplementary Material 2 [file 41598_2026_63340_MOESM2_ESM.docx]

**Supplementary Figures**

for

**Exploring imputation performance of phenotype-associated SNPs for forensic prediction models**

Zehra Köksal^a,^*, Andreas Tillmar^a,b^

^a^ Department of Biomedical and Clinical Sciences, Faculty of Health Sciences, Linköping University, SE-58183 Linköping, Sweden

^b^ Department of Forensic Genetics and Forensic Toxicology, National Board of Forensic Medicine, SE-58758 Linköping, Sweden

*Correspondence: zehra.koksal@liu.se

**
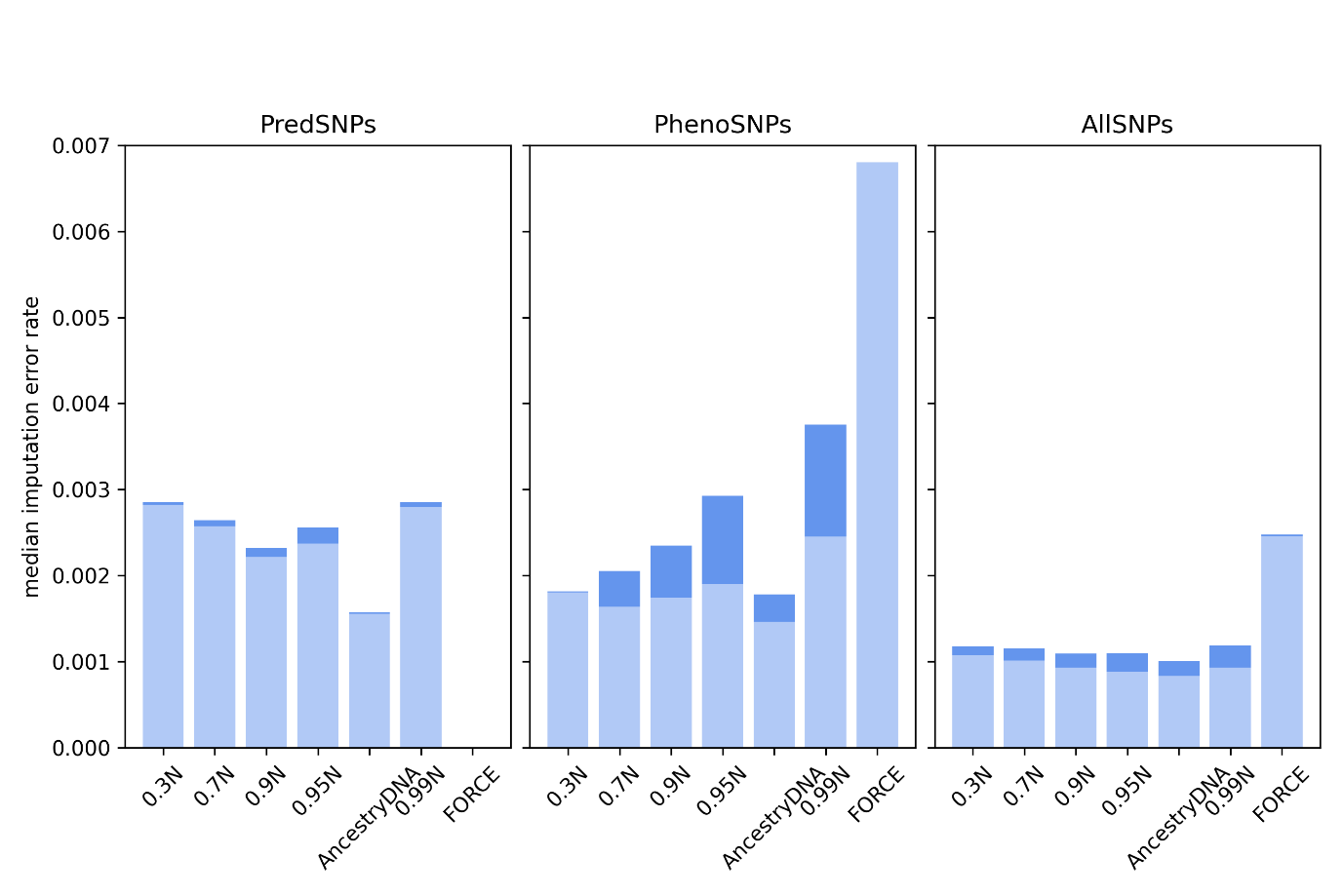
Supplementary Figure 1:** Median imputation error rates for PredSNPs, PhenoSNPs and AllSNPs using genotype probability threshold 0.99 and 7 preimputation datasets that have been unphased (background; dark blue) and maintained their true phasing (foreground; light blue) prior to imputation. For the true phasing approach, the phased data provided by the 1000 Genomes Project Consortium^1^ was used prior to subsampling for 30% to >99% missing data (0.3N to FORCE panel).


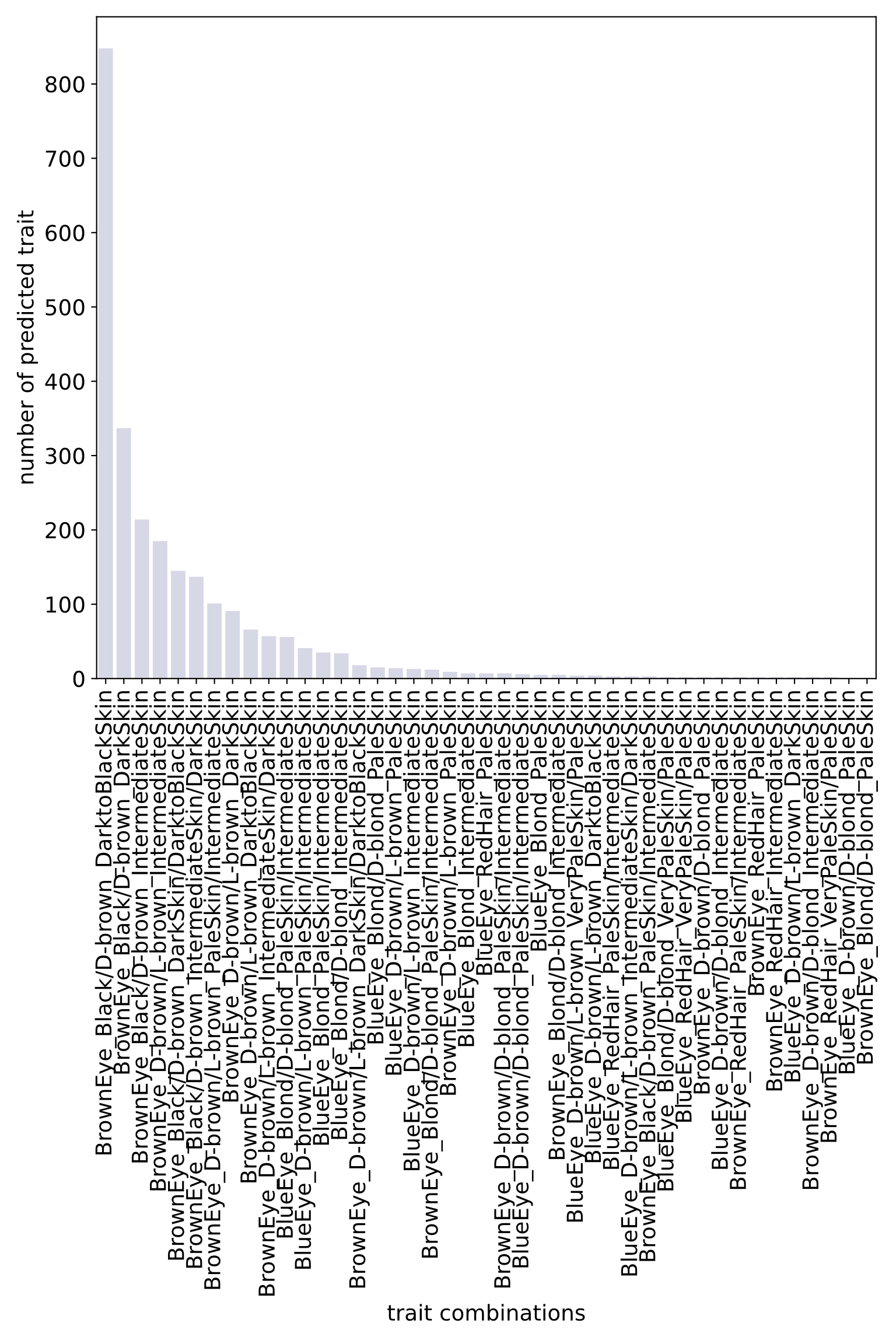


**Supplementary Figure 2:** Numbers of individuals from 1000 Genomes Project phase 3 data of each HIrisiplex-S-predicted phenotype using all 39 available SNPs.

**
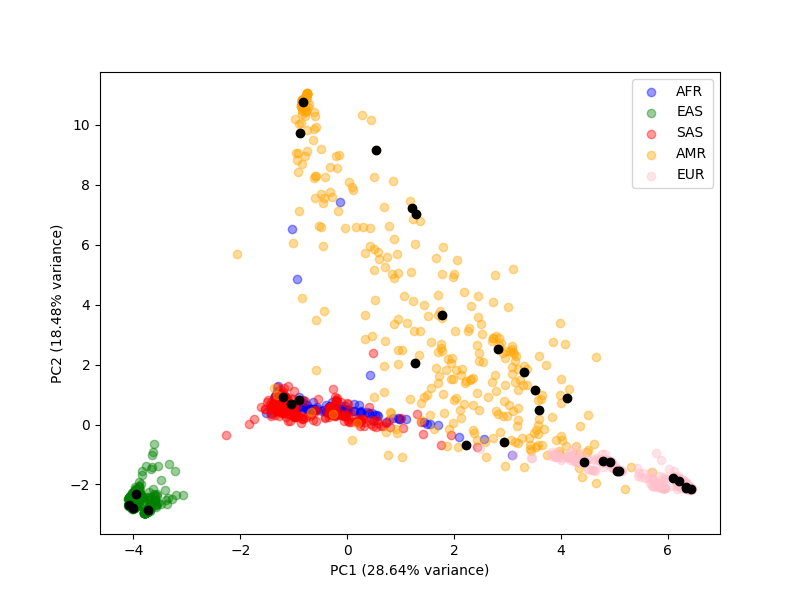
Supplementary Figure 3:** Principal Component Analysis based on the three HIrisPlex-S prediction traits and STRUCTURE cluster coefficients of the 2,473 reference panel samples coloured according to metapopulation information from 1000 Genomes Project metadata and 31 test samples coloured in black. The 31 test samples comprise of 14 AMR, 10 EUR, 4 EAS, 2 AFR and 1 SAS individuals.


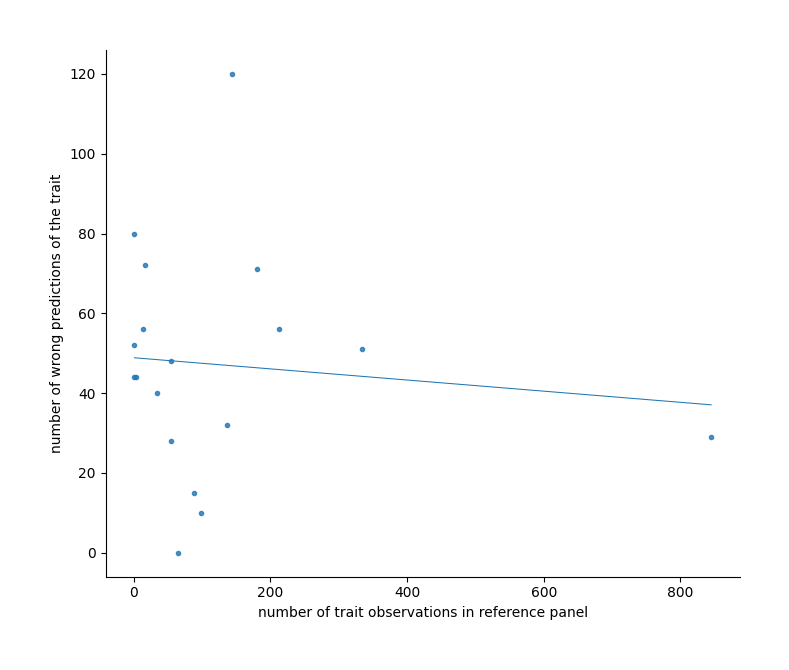
**Supplementary Figure 4:** Negative correlation of each trait’s (i.e. combination of eye, hair and skin colour) number of incorrect HIrisplex-S predictions across all 7 preimputation datasets (0.3N to FORCE) and 8 genotype probability thresholds for imputation (0.99 to 0.01) and the trait’s abundance in the reference panel. Pearson correlation: -0.100 and p-value of 6.940e-01.


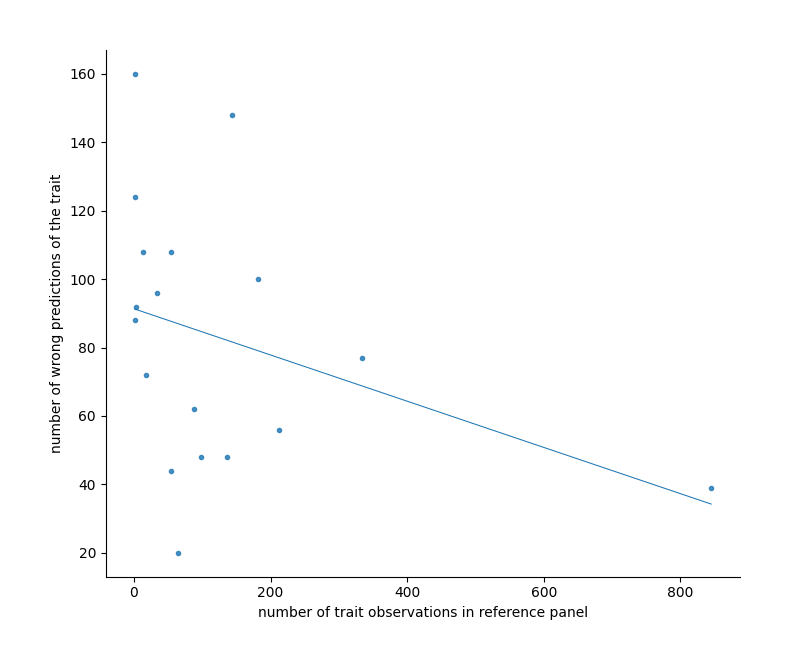


**Supplementary Figure 5:** Negative correlation of each trait’s (i.e. combination of eye, hair and skin colour) number of incorrect and inconclusive HIrisplex-S predictions across all 7 preimputation datasets (0.3N to FORCE) and 8 genotype probability thresholds for imputation (0.99 to 0.01) and the trait’s abundance in the reference panel. Pearson correlation: -0.354 and p-value of 1.493e-01.


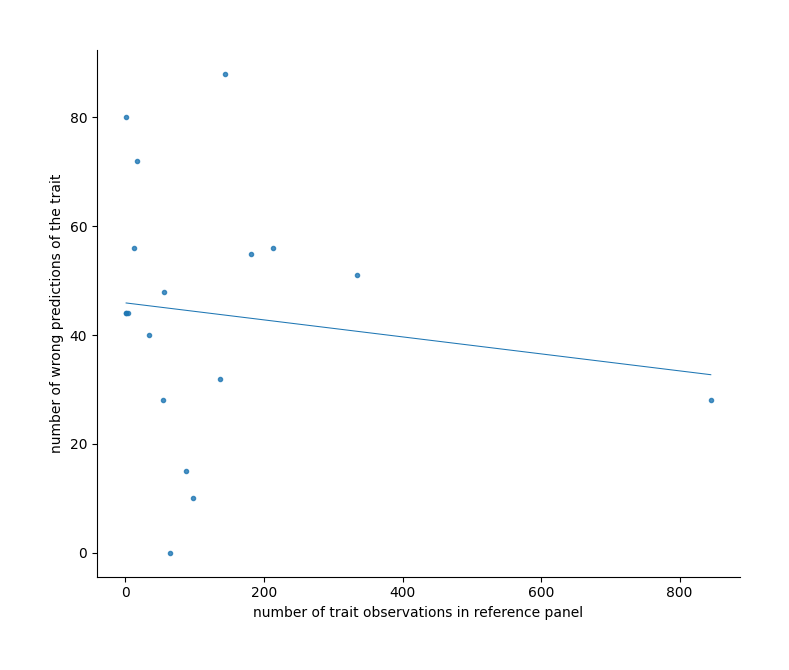


**Supplementary Figure 6:** Negative correlation of each trait’s (i.e. combination of eye, hair and skin colour) number of incorrect HIrisplex-S predictions across 5 preimputation datasets (excluding biased panel AncestryDNA and FORCE) and 8 genotype probability thresholds for imputation (0.99 to 0.01) and the trait’s abundance in the reference panel. Pearson correlation: -0.135 and p-value of 5.924e-01.


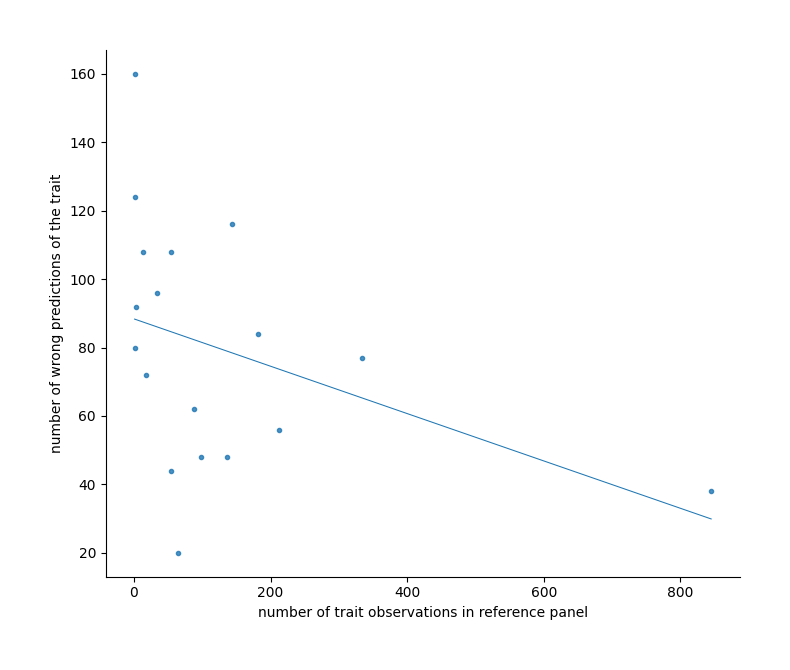


**Supplementary Figure 7:** Negative correlation of each trait’s (i.e. combination of eye, hair and skin colour) number of incorrect and inconclusive HIrisplex-S predictions across 5 preimputation datasets (excluding biased panel AncestryDNA and FORCE) and 8 genotype probability thresholds for imputation (0.99 to 0.01) and the trait’s abundance in the reference panel. Pearson correlation: -0.392 and p-value of 1.077e-01.


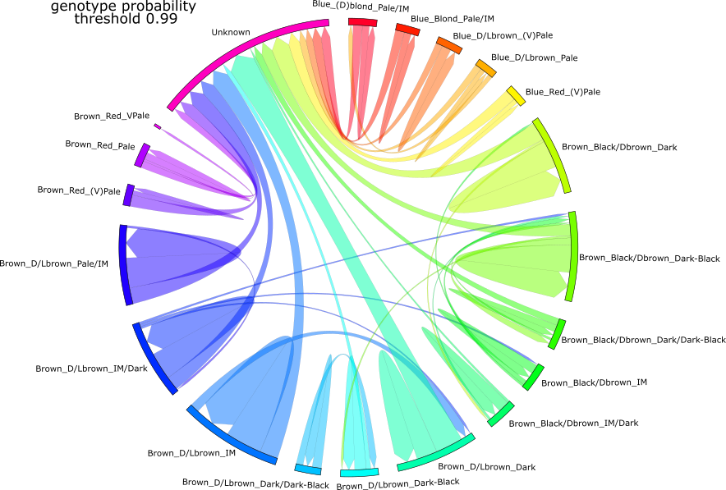


**Supplementary Figure 8:** Sankey plot with coloured sections presenting phenotypes with the true phenotypes represented as outgoing flows and their predicted phenotypes as incoming flows. Predicted samples include 31 test samples and their 7 preimputation datasets and imputation genotype threshold 0.99. The plot was generated using pyCirclize v1.10.


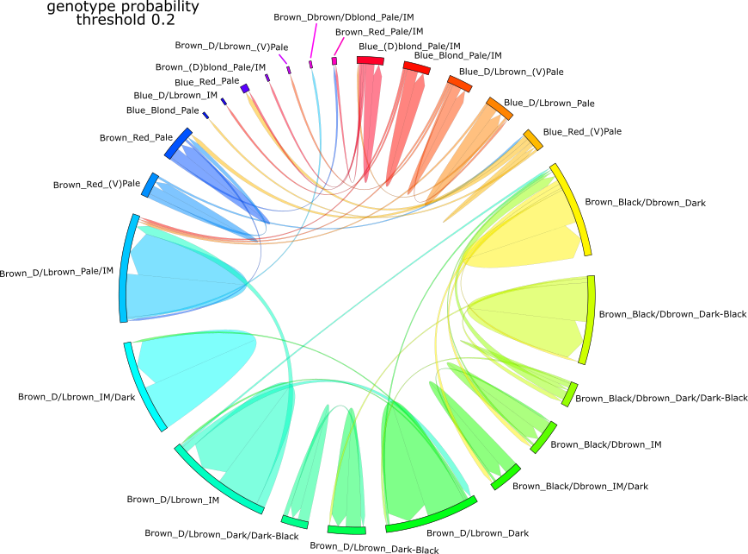


**Supplementary Figure 9:** Sankey plot with coloured sections presenting phenotypes with the true phenotypes represented as outgoing flows and their predicted phenotypes as incoming flows. Predicted samples include 31 test samples and their 7 preimputation datasets and imputation genotype threshold 0.2. The plot was generated using pyCirclize v1.10.


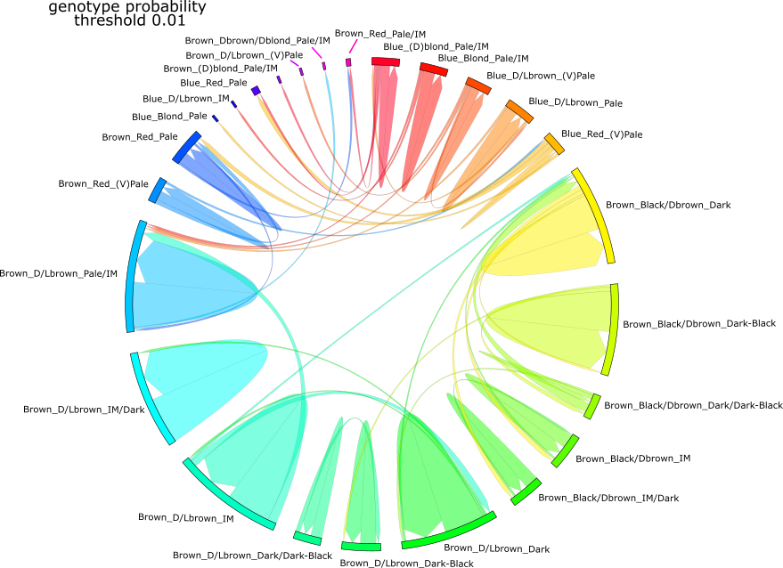


**Supplementary Figure 10:** Sankey plot with coloured sections presenting phenotypes with the true phenotypes represented as outgoing flows and their predicted phenotypes as incoming flows. Predicted samples include 31 test samples and their 7 preimputation datasets and imputation genotype threshold 0.01. The plot was generated using pyCirclize v1.10.

**
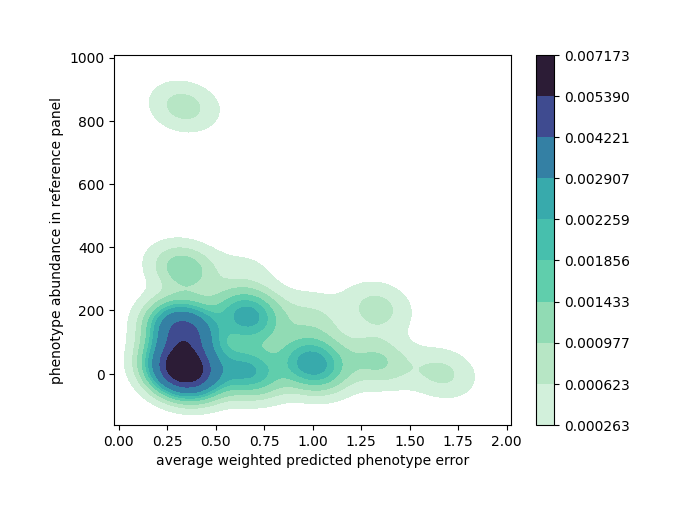
Supplementary Figure 11:** Relationship between abundance of a phenotype (combination of eye, hair and skin colour) in the imputation reference panel and the phenotype’s average weighted prediction error calculated by determining each prediction’s similarity between true and predicted phenotype. Trait similarities were quantified by ranking trait expressions and subtracting difference between true and predicted trait expression, and averaging the absolute difference across eye, hair and skin colour. The trait expression rankings follow: (A) brown (1) and blue eye (2) colour; (B) red (1) > blond (2) > dark blond (3) > dark brown/dark blond (4) > dark/light brown (5) > black/dark brown (6) hair colour; (C) very pale (1) > very pale/pale (2) > pale (3) > pale/intermediate (4) > intermediate (5) > intermediate/dark (6) > dark (7) > dark/dark-black (8) > dark-black (9) skin colour. Pearson correlation between trait abundance in the reference panel and average weighted predicted trait error shows statistically significant negative correlation (r=-0.153, p= 7.168e-06).


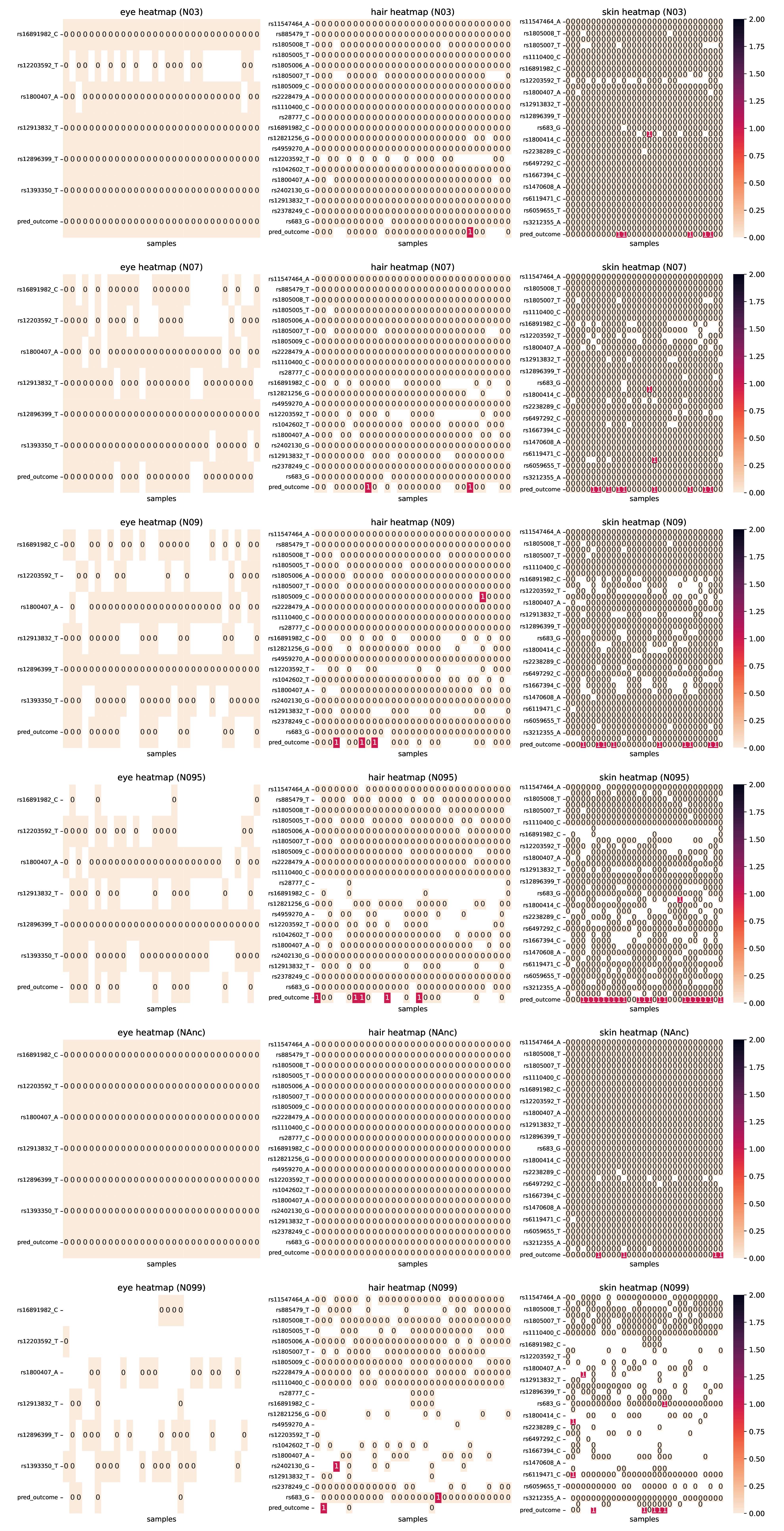


**Supplementary Figure 12:** Heatmaps of allele dosage differences (ranging from 0 to 2 or unknown) between complete dataset and imputed SNPs (0.99 genotype probability threshold) for preimputation datasets 0.3N (top panel) to 0.99N (bottom panel). The analysed SNPs represented in rows are the 6 eye colour prediction SNPs (column 1), 22 hair colour prediction SNPs (column 2), and 36 skin colour prediction SNPs (column 3) implemented in the HIrisPlex-S prediction tool. The columns present the 31 investigated samples. The last row of each subplot shows whether the sample’s trait was predicted correctly (0), wrongfully (1) or not at all (blank) when compared to the complete dataset.


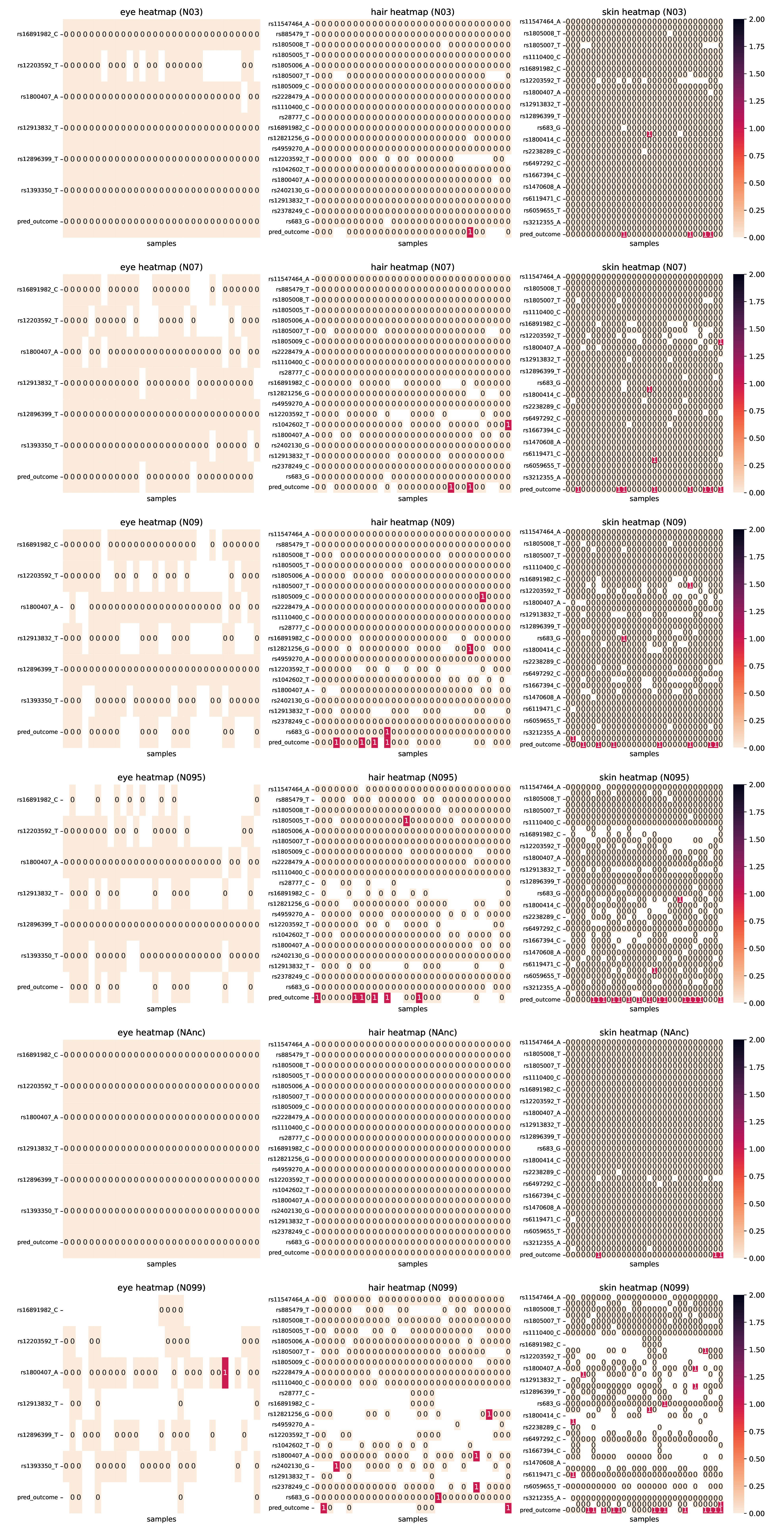


**Supplementary Figure 13:** Heatmaps of allele dosage differences (ranging from 0 to 2 or unknown) between complete dataset and imputed SNPs (0.95 genotype probability threshold) for preimputation datasets 0.3N (top panel) to 0.99N (bottom panel). The analysed SNPs represented in rows are the 6 eye colour prediction SNPs (column 1), 22 hair colour prediction SNPs (column 2), and 36 skin colour prediction SNPs (column 3) implemented in the HIrisPlex-S prediction tool. The columns present the 31 investigated samples. The last row of each subplot shows whether the sample’s trait was predicted correctly (0), wrongfully (1) or not at all (blank) when compared to the complete dataset.


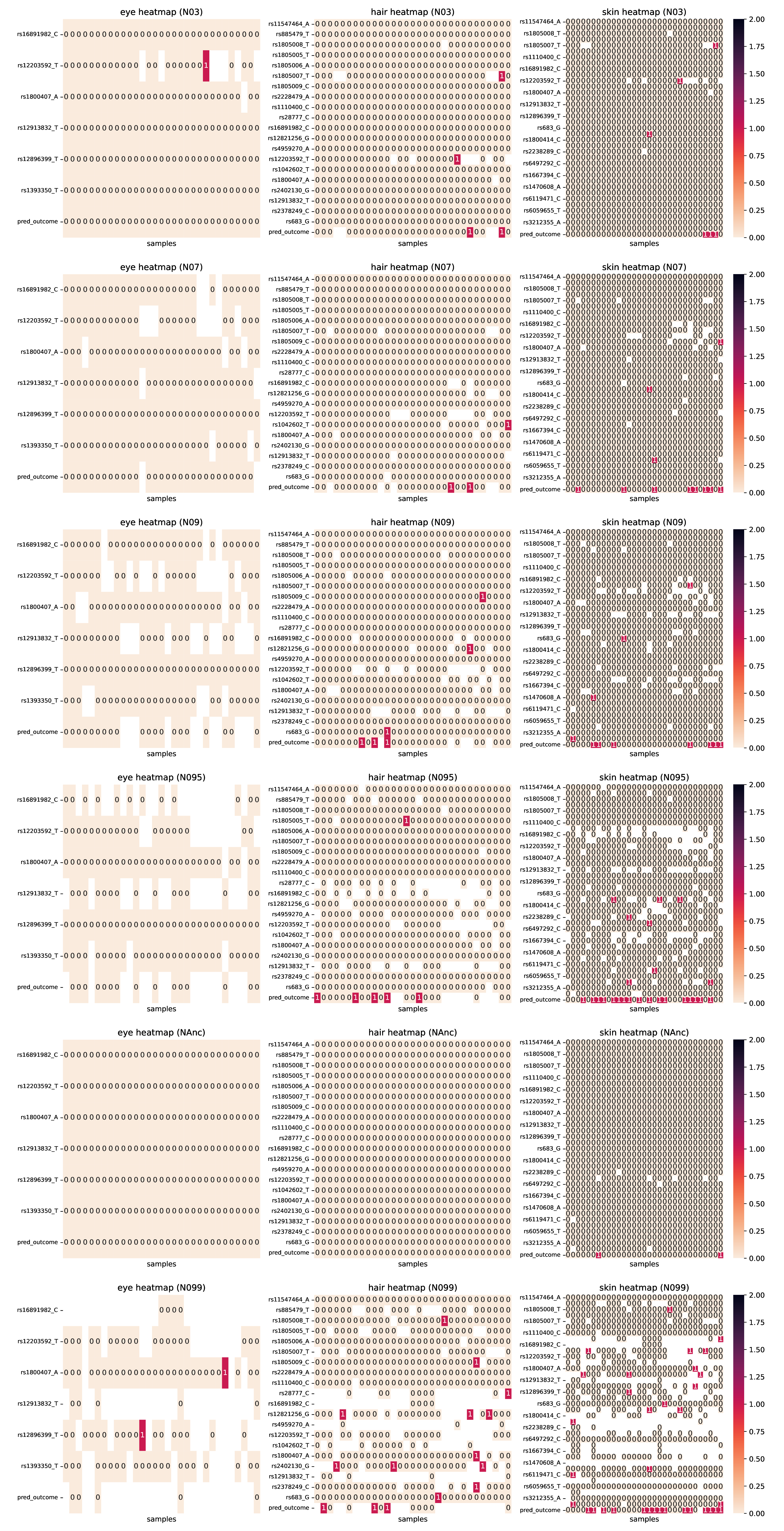


**Supplementary Figure 14:** Heatmaps of allele dosage differences (ranging from 0 to 2 or unknown) between complete dataset and imputed SNPs (0.9 genotype probability threshold) for preimputation datasets 0.3N (top panel) to 0.99N (bottom panel). The analysed SNPs represented in rows are the 6 eye colour prediction SNPs (column 1), 22 hair colour prediction SNPs (column 2), and 36 skin colour prediction SNPs (column 3) implemented in the HIrisPlex-S prediction tool. The columns present the 31 investigated samples. The last row of each subplot shows whether the sample’s trait was predicted correctly (0), wrongfully (1) or not at all (blank) when compared to the complete dataset.


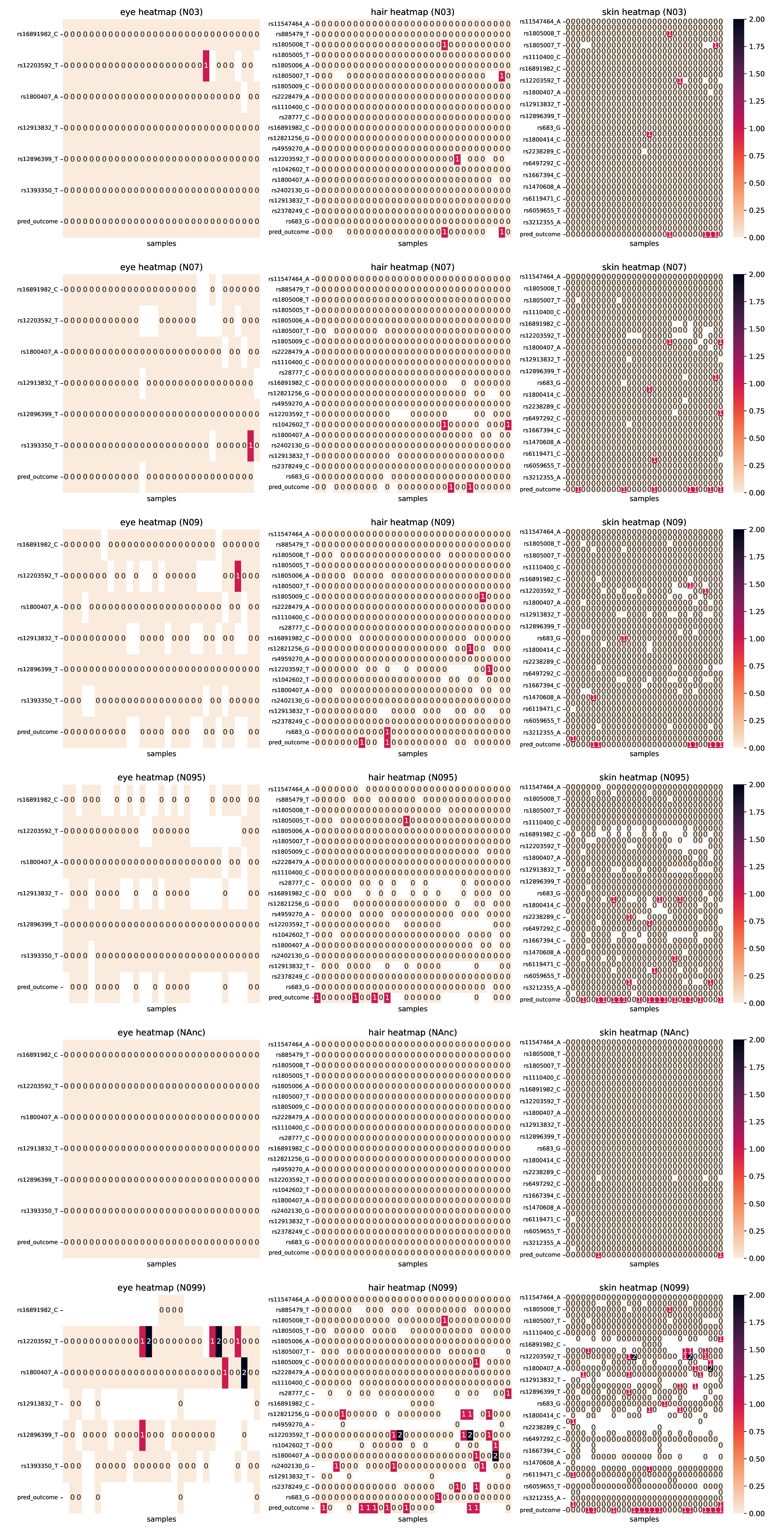


**Supplementary Figure 15:** Heatmaps of allele dosage differences (ranging from 0 to 2 or unknown) between complete dataset and imputed SNPs (0.85 genotype probability threshold) for preimputation datasets 0.3N (top panel) to 0.99N (bottom panel). The analysed SNPs represented in rows are the 6 eye colour prediction SNPs (column 1), 22 hair colour prediction SNPs (column 2), and 36 skin colour prediction SNPs (column 3) implemented in the HIrisPlex-S prediction tool. The columns present the 31 investigated samples. The last row of each subplot shows whether the sample’s trait was predicted correctly (0), wrongfully (1) or not at all (blank) when compared to the complete dataset.


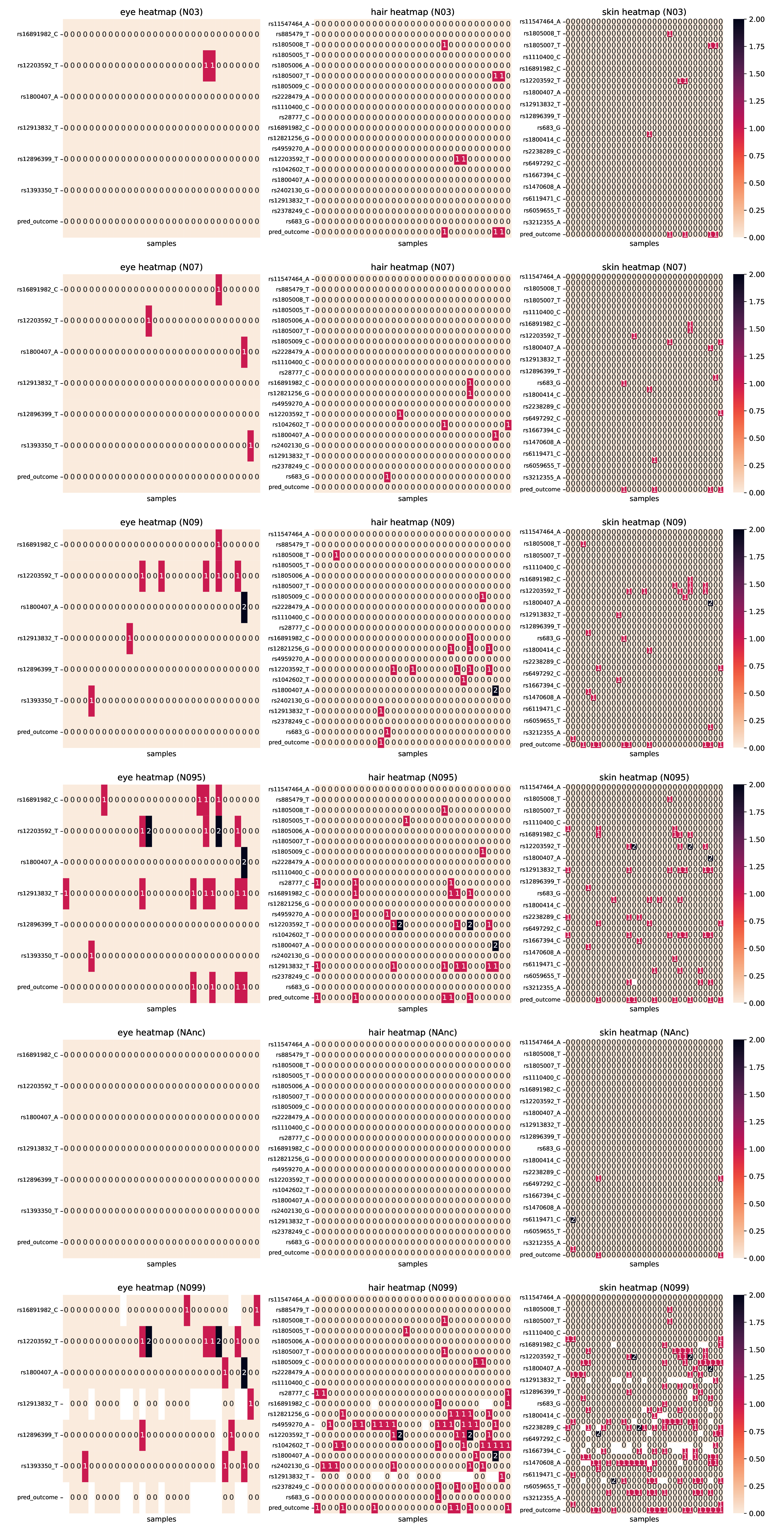


**Supplementary Figure 16:** Heatmaps of allele dosage differences (ranging from 0 to 2 or unknown) between complete dataset and imputed SNPs (0.5 genotype probability threshold) for preimputation datasets 0.3N (top panel) to 0.99N (bottom panel). The analysed SNPs represented in rows are the 6 eye colour prediction SNPs (column 1), 22 hair colour prediction SNPs (column 2), and 36 skin colour prediction SNPs (column 3) implemented in the HIrisPlex-S prediction tool. The columns present the 31 investigated samples. The last row of each subplot shows whether the sample’s trait was predicted correctly (0), wrongfully (1) or not at all (blank) when compared to the complete dataset.


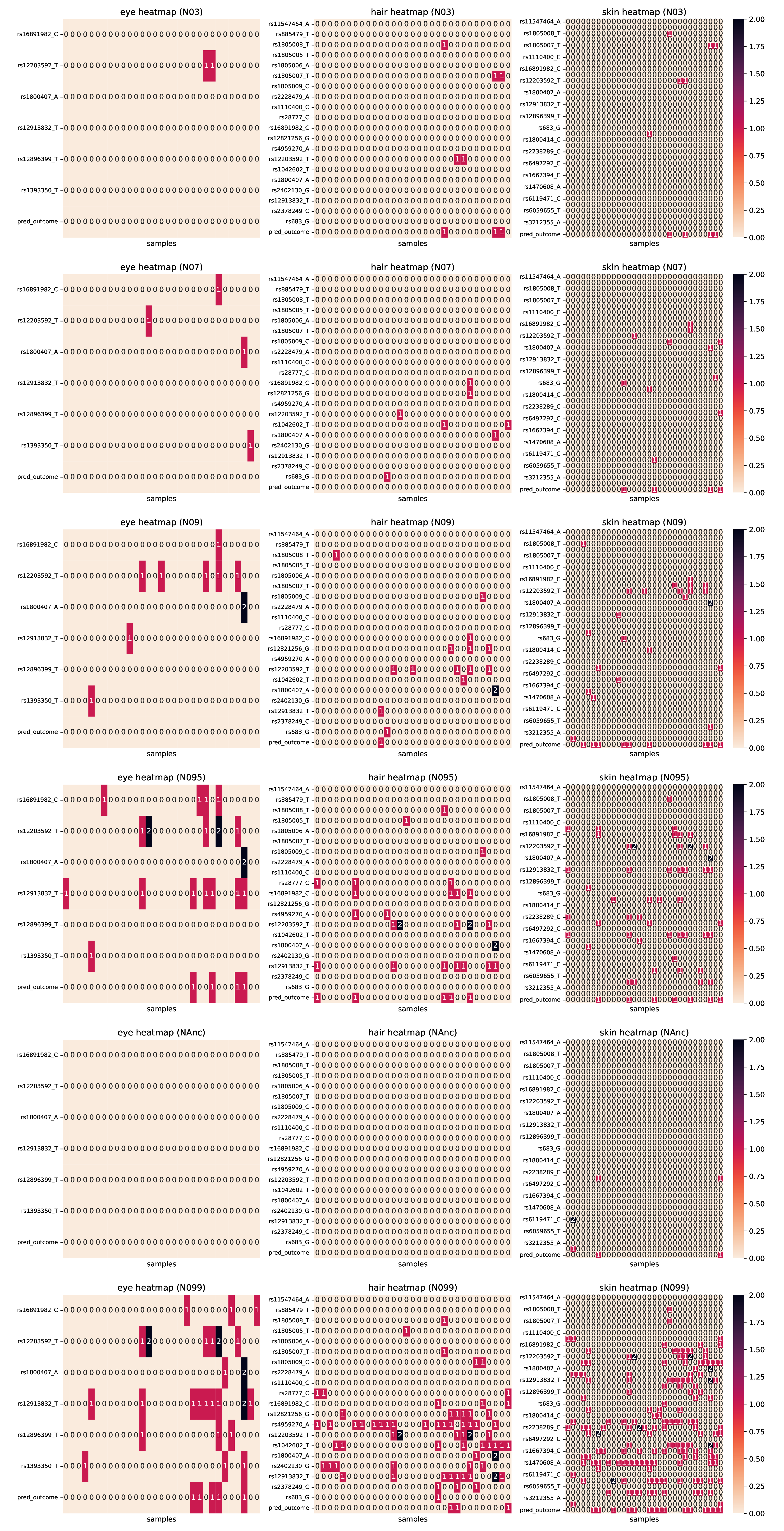


**Supplementary Figure 17:** Heatmaps of allele dosage differences (ranging from 0 to 2 or unknown) between complete dataset and imputed SNPs (0.2 genotype probability threshold) for preimputation datasets 0.3N (top panel) to 0.99N (bottom panel). The analysed SNPs represented in rows are the 6 eye colour prediction SNPs (column 1), 22 hair colour prediction SNPs (column 2), and 36 skin colour prediction SNPs (column 3) implemented in the HIrisPlex-S prediction tool. The columns present the 31 investigated samples. The last row of each subplot shows whether the sample’s trait was predicted correctly (0), wrongfully (1) or not at all (blank) when compared to the complete dataset.


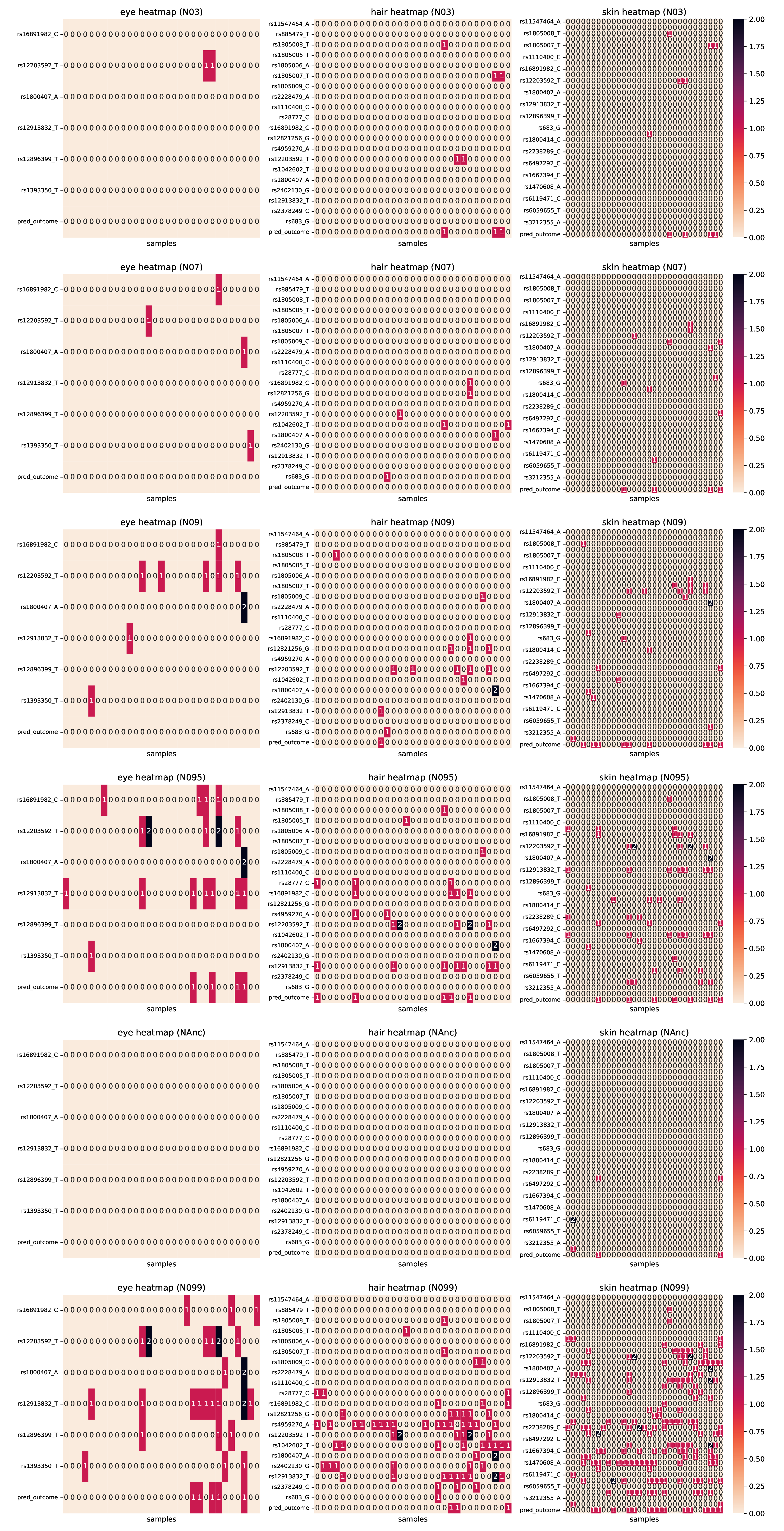


**Supplementary Figure 18:** Heatmaps of allele dosage differences (ranging from 0 to 2 or unknown) between complete dataset and imputed SNPs (0.1 genotype probability threshold) for preimputation datasets 0.3N (top panel) to 0.99N (bottom panel). The analysed SNPs represented in rows are the 6 eye colour prediction SNPs (column 1), 22 hair colour prediction SNPs (column 2), and 36 skin colour prediction SNPs (column 3) implemented in the HIrisPlex-S prediction tool. The columns present the 31 investigated samples. The last row of each subplot shows whether the sample’s trait was predicted correctly (0), wrongfully (1) or not at all (blank) when compared to the complete dataset.


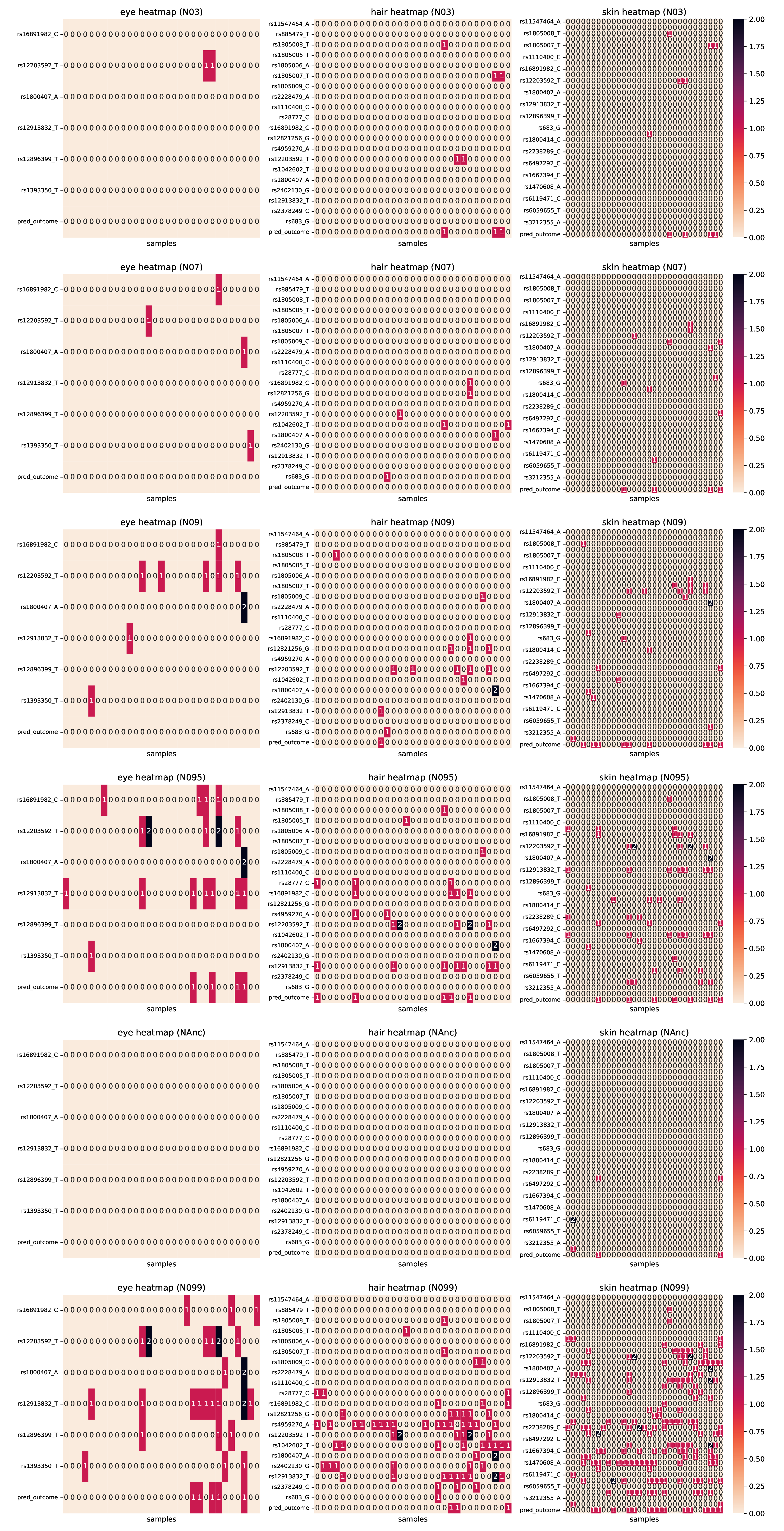


**Supplementary Figure 19:** Heatmaps of allele dosage differences (ranging from 0 to 2 or unknown) between complete dataset and imputed SNPs (0.01 genotype probability threshold) for preimputation datasets 0.3N (top panel) to 0.99N (bottom panel). The analysed SNPs represented in rows are the 6 eye colour prediction SNPs (column 1), 22 hair colour prediction SNPs (column 2), and 36 skin colour prediction SNPs (column 3) implemented in the HIrisPlex-S prediction tool. The columns present the 31 investigated samples. The last row of each subplot shows whether the sample’s trait was predicted correctly (0), wrongfully (1) or not at all (blank) when compared to the complete dataset.


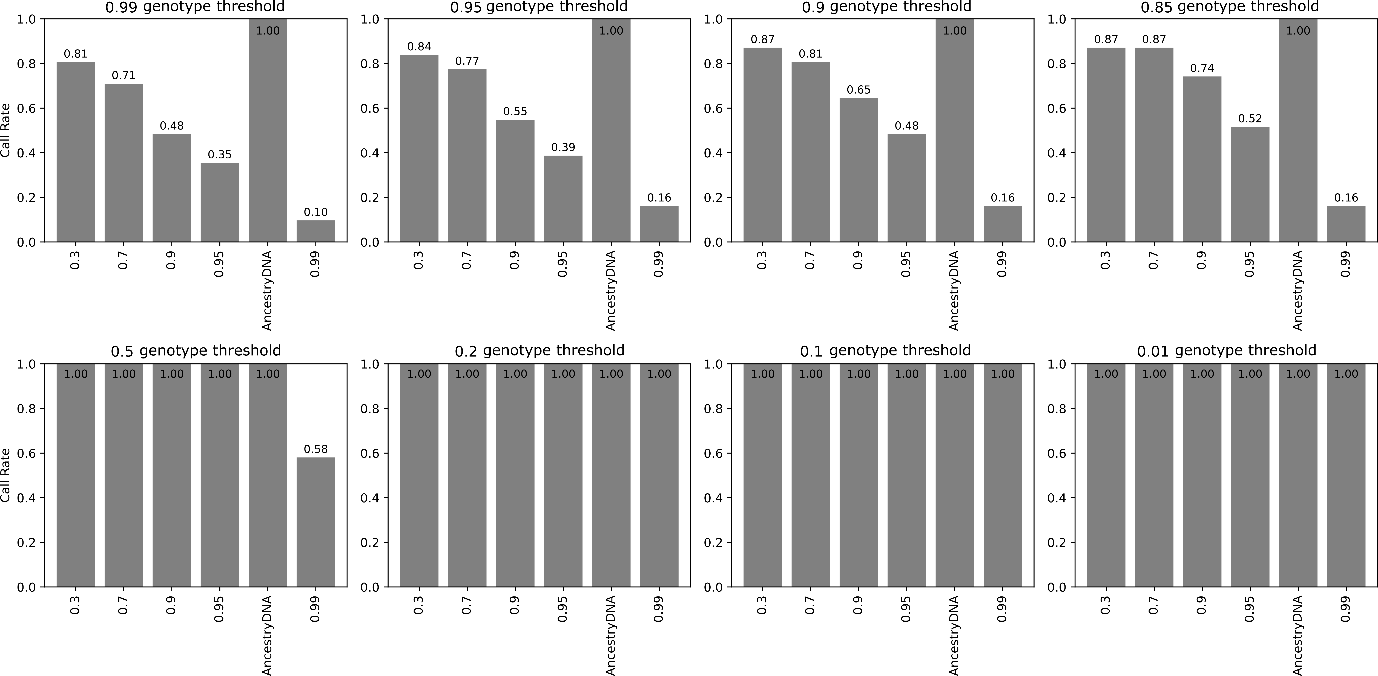
**Supplementary Figure 20:** HIrisPlex-S prediction call rate for 31 samples in 6 preimputation datasets from 30% to 99% missing data for 8 genotype probability thresholds from 0.99 to 0.01.


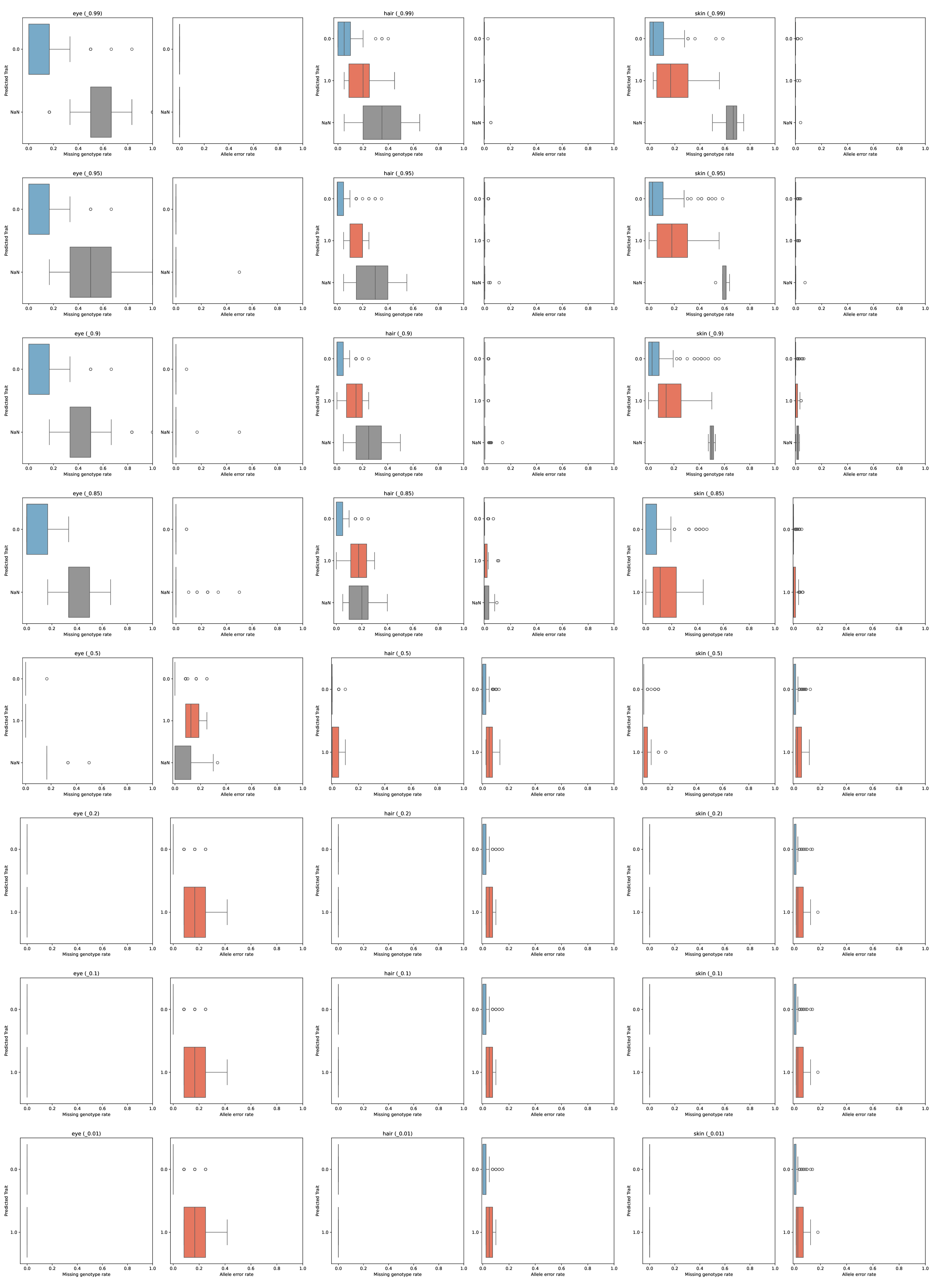


**Supplementary Figure 21:** Distribution of per-sample and post-imputation missing genotype rates (left x axes) and allele error rates (right x axes) for SNP genotypes incorporated in predicting eye colour (column 1), hair colour (column 2) and skin colour (column 3). The corresponding individual’s trait was predicted correctly (prediction trait: 0; blue), incorrectly (prediction trait: 1; red) or was undetermined (prediction trait: NaN; grey). Each row represents imputations with different genotype probability thresholds from (top panel) 0.99 to (bottom panel) 0.01.

**
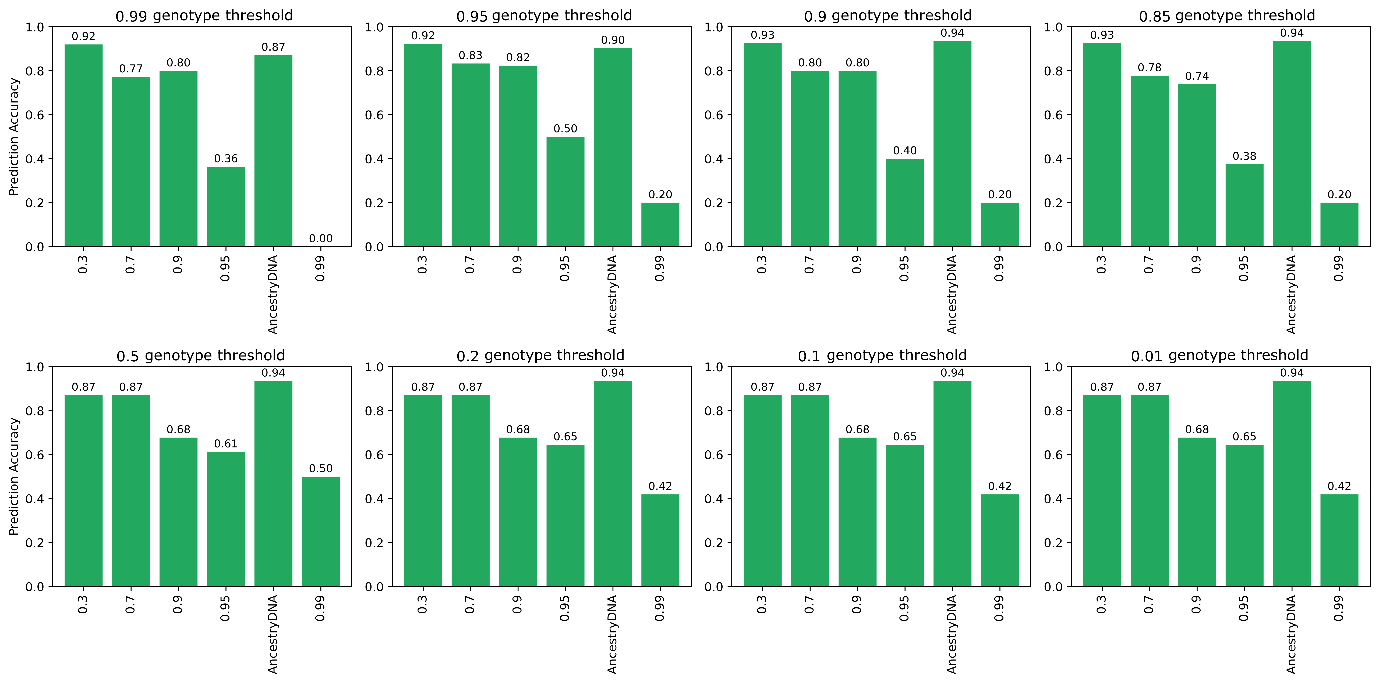
Supplementary Figure 22:** HIrisPlex-S prediction accuracy among predicted phenotypes for 31 samples in 6 preimputation datasets from 30% to 99% missing data for 8 genotype probability thresholds from 0.99 to 0.01.


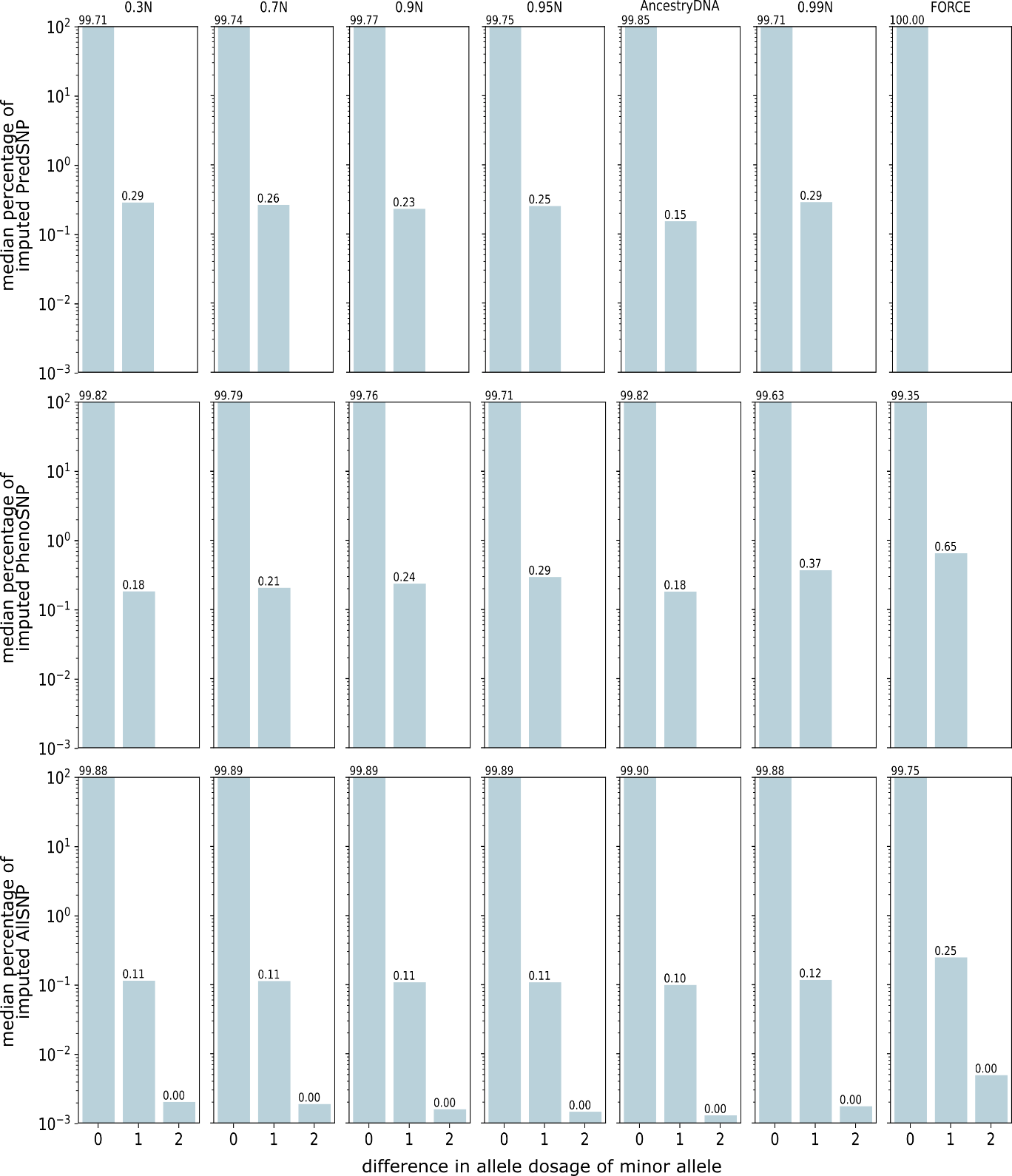
**Supplementary Figure 23:** Median percentage of allele dosage difference of the minor allele between the true and imputed PredSNP (upper panel), PhenoSNP (middle panel), and AllSNP (bottom panel) genotypes across 31 individuals of seven different preimputation datasets 0.3N to FORCE panel (columns).


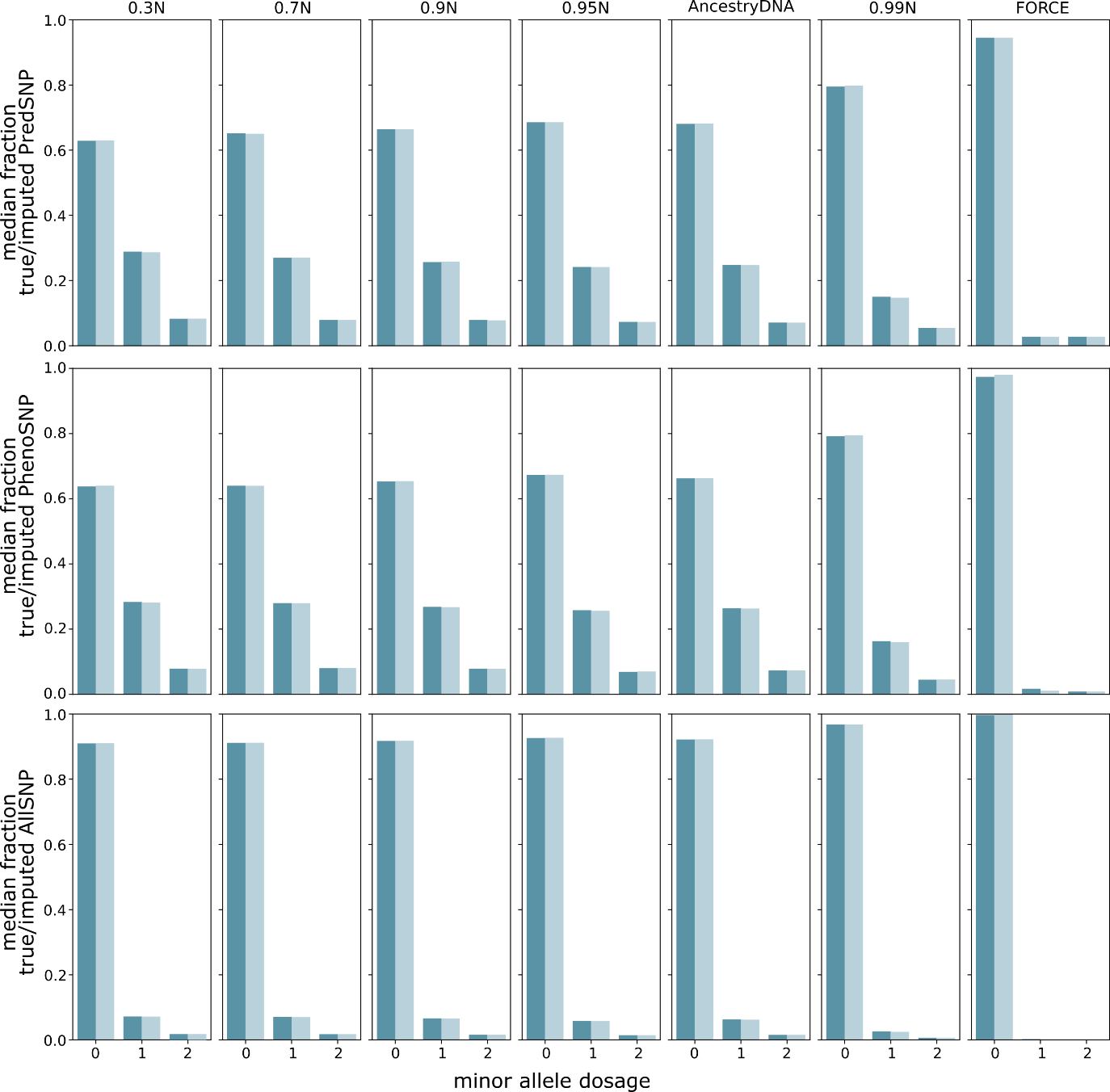


**Supplementary Figure 24:** Median fraction of PredSNPs (upper panel), PhenoSNPs (middle panel), and AllSNPs (bottom panel) with minor allele dosage of 0, 1 or 2 in true (dark blue bars) and imputed (light blue bars) SNP genotypes considering only imputed SNP genotypes using 7 preimputation datasets 0.3N to FORCE panel (columns).


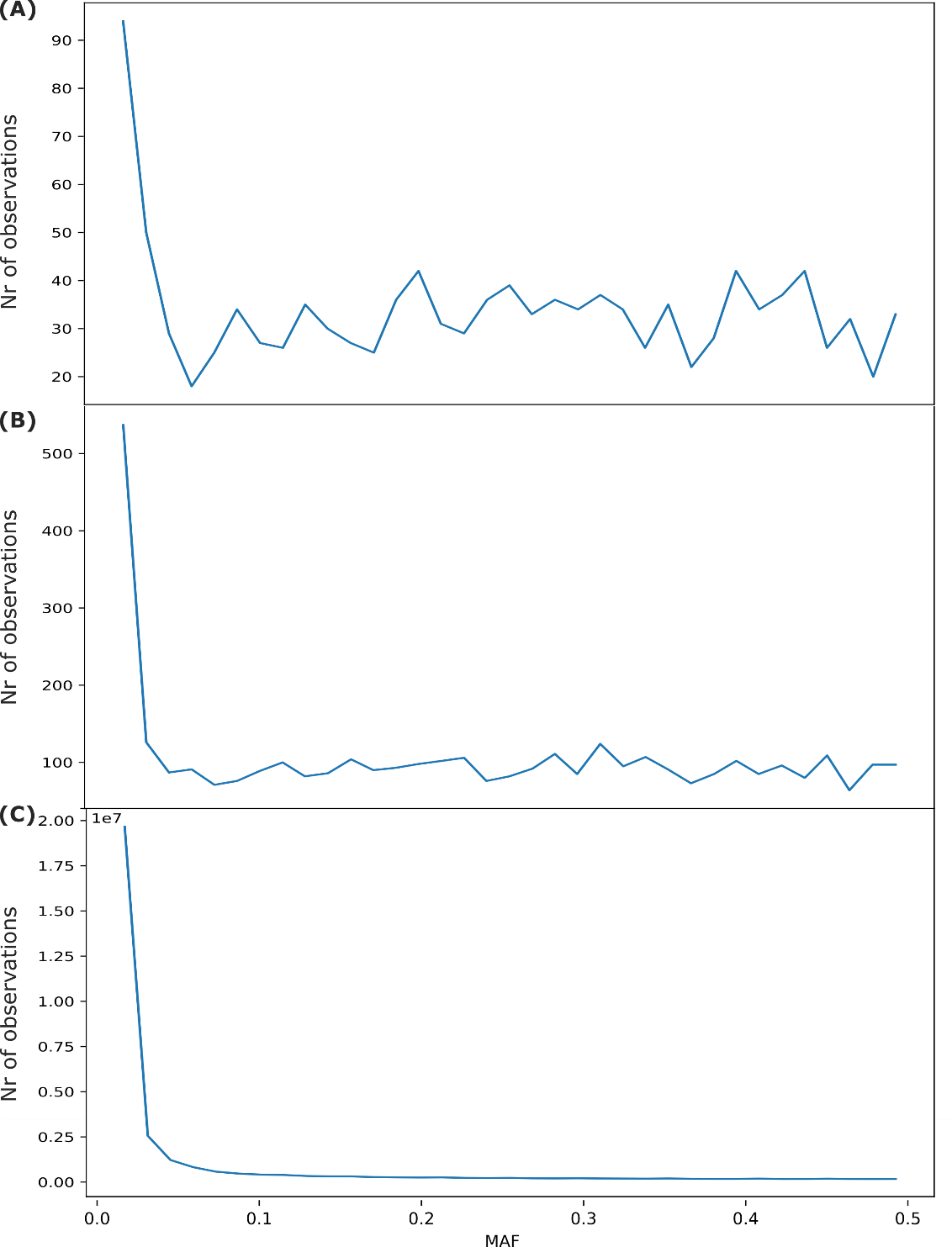


**Supplementary Figure 25:** Distribution of minor allele frequencies (MAF) for (A) PredSNPs, (B) PhenoSNPs and (C) AllSNPs.


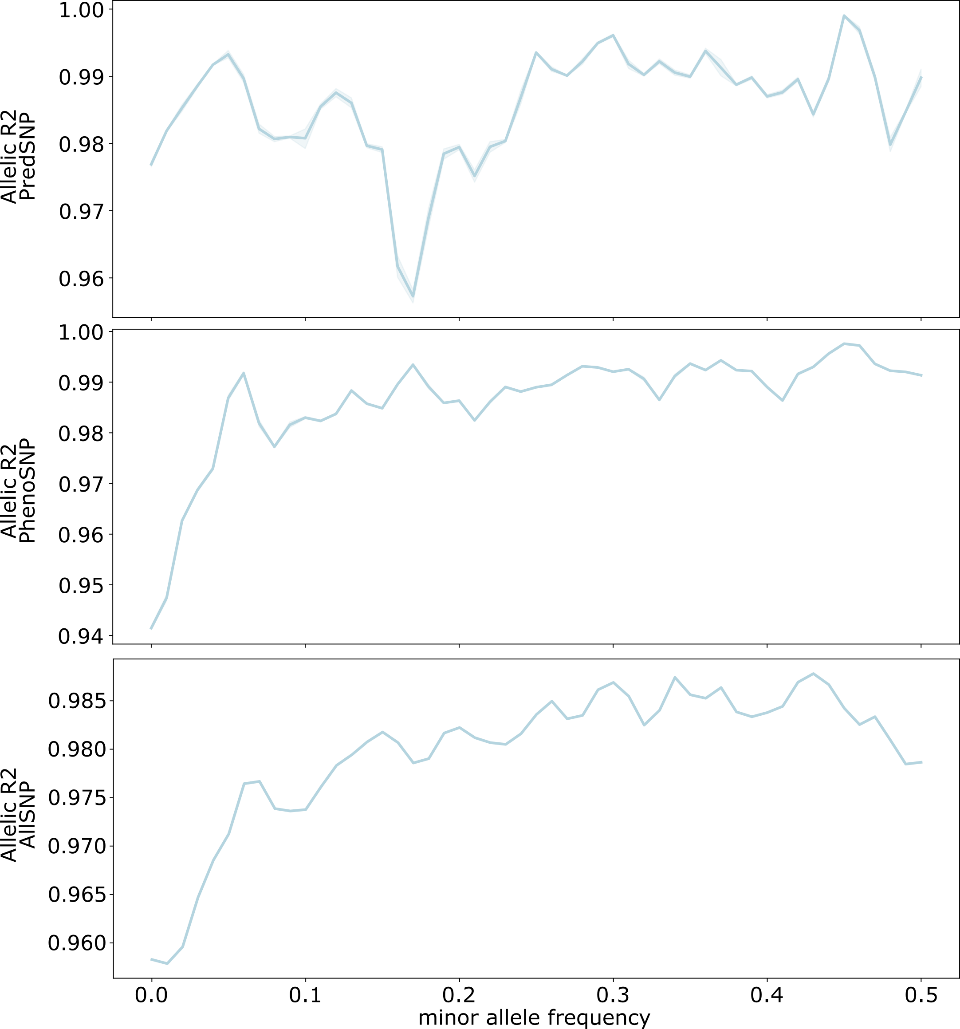


**Supplementary Figure 26:** Squared correlation between the true and imputed minor allele dosage across all PredSNPs (top panel), PhenoSNPs (middle panel), or AllSNPs (bottom panel) with the same minor allele frequency (MAF) with 5 decimal values. To reduce local R^2^ variability, all R^2^ values per MAF were averaged across all SNPs with ±0.01 MAF across the different preimputation datasets. For the genotype imputation, a threshold of 0.99 was used. Please note that the high imputation accuracy of SNPs with very low MAFs are smoothed out and thus not represented in the plot.

**Reference**

1. Auton, A. *et al.* A global reference for human genetic variation. *Nature* **526**, 68–74 (2015).
